# Artificial intelligence in the identification and prediction of adverse transfusion reactions(ATRs) and implications for clinical management: a systematic review of models and applications

**DOI:** 10.1186/s12911-025-03232-z

**Published:** 2025-10-28

**Authors:** Mahdie ShojaeiBaghini, Mohammad Mehdi Ghaemi, Alihasan Ahmadipour

**Affiliations:** 1 Medical Informatics Research Center, Institute for Futures Studies in Health, Kerman University of Medical Sciences, Kerman, Iran; 2 Modeling in Health Research Center, Institute for Futures Studies in Health, Kerman University of Medical Sciences, Kerman, Iran; 3 Faculty of Management and Medical Information Sciences, Kerman University of Medical Sciences, Kerman, Iran

**Keywords:** Adverse effects, Adverse transfusion reactions (ATRs), Artificial intelligence (AI), Blood transfusion, Hemovigilance, Patient safety

## Abstract

**Background:**

Despite advances in patient safety, adverse transfusion reactions (ATRs) continue to occur in clinical settings and remain a primary focus of hospital hemovigilance committees. Artificial intelligence (AI) has emerged as a promising tool for detecting and preventing these complications. The objective of this study is to synthesize the evidence on the applications of AI in identifying and predicting ATRs, and to examine the existing evidence regarding the feasibility and effectiveness of employing these tools in active clinical management.

**Methods:**

This systematic review (SR) was conducted according to the PRISMA 2020 guidelines. English-language articles published within the last decade, focusing on the application of AI in the Identification and Prediction of ATRs and Implications for Clinical Management, were retrieved from the PubMed, Scopus, and Web of Science databases and subsequently analyzed. The quality of the included studies was assessed using the QUADAS-AI tool, and the findings are presented descriptively.

**Results:**

This SR showed that in the 24 included studies, AI models were primarily applied across four main focal areas: transfusion risks and outcomes, risk and moderating factors, transfusion volume and intensity, and classification and extraction of ATRs. In the included studies, the most essential model evaluation metrics were AUROC and Sensitivity, each reported in nine studies, followed by Accuracy and F1-Score, each reported in five studies. Among the studies, the Random Forest (RF) model was used more frequently than other models. Moreover, none explicitly addressed the development, implementation, or clinical evaluation of an active management system based on AI. Clinically, most studies focused on transfusion-related complications such as mortality, bleeding, and morbidity. The majority of the studies were conducted in the field of Hematology, followed by cardiology, surgery, and ICU units.

**Conclusions:**

Based on the interpretation of results, individual patient factors and transfusion volume play a pivotal role in the occurrence of ATRs. Implementing safe transfusion strategies, including the use of clinical decision support systems (CDSS) integrated with electronic health records (EHR) and personalized medicine approaches, alongside adherence to ethical considerations and patient privacy protection, is essential in future research. This study also identified two significant research gaps: first, the lack of research on the implementation or clinical evaluation of AI-based active management systems for ATRs; and second, the analysis of population groups revealed that research has been predominantly focused on adults, highlighting a gap concerning vulnerable populations, particularly pediatric patients.

**Supplementary Information:**

The online version contains supplementary material available at 10.1186/s12911-025-03232-z.

## Background

### Rationale of the study

Adverse transfusion reactions (ATRs) are potential risks inherently linked to the transfusion of blood and its components, and a thorough understanding of these reactions is essential to enhance transfusion safety [1]. Despite significant improvements in increasing patient safety, ATRs are harmful or unintended responses that occur in a patient during or after a blood transfusion. They are of primary concern for blood transfusion systems. ATRs are generally classified into infectious and non-infectious types, with Non-Infectious ATRs (NIATRs) being more prevalent [2]. NIATRs are categorized based on the time of onset into acute and delayed types, and according to the likely underlying mechanism into immune-mediated and non-immune-mediated subgroups [3]. ATRs have substantial economic and clinical consequences. It can increase healthcare costs due to the requirement for extra examinations, treatments, and increased allocation of resources. Even moderate ATRs may cause an economic burden on the healthcare system, presenting opportunities for cost savings and enhancements in therapeutic procedures [4]. ATRs in clinical settings may lead to serious complications such as transfusion-associated circulatory overload, acute lung injury caused by blood transfusions, allergic responses, and acute hemolytic reactions [1]. Based on studies, the rates of ATRs differ significantly among countries, with observations of 0.7% in Colombia [5], 0.14% in India [6], 2.99% in Burkina Faso [7], and 4% in Iran [8]. The high incidence of ATRs highlights the growing need for effective strategies to ensure timely identification and prevention. One of the main objectives of hemovigilance experts (Hemovigilance experts are professionals responsible for monitoring, identifying, reporting, and preventing complications and ATR processes to ensure patient safety) is to reduce the incidence of ATRs through timely and effective identification.

Artificial Intelligence (AI), as an advanced technology, has emerged as a promising and effective tool for the identification and mitigation of ATRs. AI models possess high capability in classifying and identifying ATRs and can perform comparably to transfusion medicine specialists [9]. These models enable the prediction, detection, and management of ATRs, contributing to the reduction of complications and enhancement of patient safety. From a predictive and detection perspective, the primary goal is to prevent and reduce the occurrence of ATRs. In contrast, from a management standpoint, ATRs should be assessed simultaneously and immediately at the point of care (POC), with necessary interventions implemented to mitigate complications and improve treatment [10].

Numerous studies have shown that AI models accurately identify and detect ATRs while demonstrating high innovation and effectiveness. The study by Murphree et al. demonstrated that AI has significant potential in predicting transfusion-related complications such as transfusion-associated circulatory overload (TACO: a condition caused by excessive blood transfusion volume, leading to breathing difficulty and swelling) and transfusion-related acute lung injury (TRALI: a sudden lung injury after a blood transfusion, causing severe breathing problems) [11]. Additionally, another study showed that the use of AI is increasing in areas such as blood group compatibility, complication prediction, and identification of biomarkers related to transfusion [12]. From an operational perspective, the model’s effectiveness is primarily influenced by the “Garbage In, Garbage Out” principle [13], with accuracy being contingent upon the quality of its input data. On the other hand, ensuring patient safety during the blood transfusion is necessary and unavoidable. It suggests that the essential data sources be fully prepared and organized before implementation to improve model accuracy and operational efficiency.

Despite the widespread use of AI in investigating ATRs, a review of databases and journals such as Vox Sanguinis, Transfusion, and International Prospective Register of Systematic Reviews (PROSPERO) revealed that no systematic review(SR) exists on the identification, prediction, and clinical management of ATRs. An SR can fill this gap by examining existing models, assessing their effectiveness and challenges, and identifying their practical applications. This approach allows for better comparison between different methods and provides a clearer direction for future research and clinical use. Ultimately, such a review can help transfusion specialists and healthcare providers make better use of AI technologies to improve patient safety and reduce complications related to blood transfusions.

### Objective

The objective of this study is to synthesize the evidence on the applications of AI in identifying and predicting ATRs, and to examine the existing evidence regarding the feasibility and effectiveness of employing these tools in active clinical management. The findings of this research aim to guide future studies and provide a framework for enhancing the application of AI in blood transfusion services and improving patient safety.

## Method

### Study methodology

#### Study design

This SR not only examines predictive models for ATRs but also includes studies on the development and evaluation of AI-based active management systems. The review was conducted according to the Preferred Reporting Items for Systematic Reviews and Meta-Analyses (PRISMA, 2020 guidelines [14]. It was based on a protocol registered in PROSPERO under the identifier CRD420251019252.

#### Inclusion and exclusion criteria

The inclusion criteria consist of English-language studies from the past decade focusing on the application of AI in the identification and prediction of ATRs and clinical management, with both abstract and full text accessible. Exclusion criteria include studies that fall outside the specified time frame, lack full-text availability, are unrelated to the study topic, or are published in languages other than English. Studies with weak methodology, a lack of proper model evaluation, or those that are non-research articles, such as reviews, letters, and book chapters, are also excluded. Additionally, studies that had not undergone peer review and those that were presented in conference proceedings were also excluded.

#### Information sources

This SR utilized Web of Science, PubMed, and Scopus as the primary information sources. Following the set inclusion criteria, the search was conducted from January 1, 2015, to August 6, 2025. Given the increase in AI-based studies over the past decade [15, 16], the focus has been on studies from the last ten years.

#### Search strategy

The SRs search method was carefully designed to effectively and completely discover publications relevant to AI and adverse effects associated with blood transfusions. The strategy included keywords pertinent to the study objective, such as “Transfusion Reaction”, “Artificial Intelligence”, and “Blood Transfusion”. These keywords were combined with Medical Subject Headings (MeSH) to enhance the search’s accuracy and scope. The search strategy for each information source is provided in Table A1 in the Appendix.

#### Study selection

Based on the studies that were determined, key information such as titles, abstracts, keywords, author names, institutional affiliations, journal titles, and publication years was collected. This information was then imported into EndNote reference management software (version 2021) for effective data extraction and organization. After removing duplicates, two authors (AH-A, M-SB) independently reviewed the titles and abstracts. Discrepancies were resolved through discussion or, if necessary, by consulting a third author (MM- GH). Then, obtained full-text articles for studies deemed relevant. Studies that did not meet the inclusion criteria after full-text review were excluded, and the reasons for exclusion are presented in Table A2 in the Appendix.

### Data collection and analysis

#### Data items

After the final studies were selected, two authors (AH-A and M-SB) independently extracted key data using a data extraction form in Microsoft Word 2021. Any disagreements regarding the data were resolved by a third author (MM-GH). Extracted data include the study identification number, first author’s name (as reference), publication year, study design, country of origin, study objectives, key findings, Type of Complications, Clinical Units, AI model, population group, Model Performance Metrics and Value, and data type. In studies where more than one AI model was developed, the model with the best overall performance was selected for analysis. The results of the data extraction process are presented in Table A3 in the Appendix.

#### Quality assessment

To assess the risk of bias and applicability concerns in the included studies, the Quality Assessment of Diagnostic Accuracy Studies -AI (QUADAS-AI) tool, specifically designed for assessment of the quality of diagnostic accuracy studies involving AI, was used [17]. Two authors independently performed the assessment, and any disagreements were resolved through consultation with a third author. The results of the assessment are presented in Table A4 in the Appendix.

#### Analysis

A meta-analysis was not feasible due to heterogeneity in study designs, AI models, and outcome metrics. Results were synthesized narratively and presented using descriptive statistics. Tables and figures were generated using Datawrapper [18], highlighting key study characteristics, types of AI models used, and their reported effectiveness.

## Results

### Descriptive overview of the included studies and quality assessment

#### Frequency of included and excluded studies

**Fig. 1:**
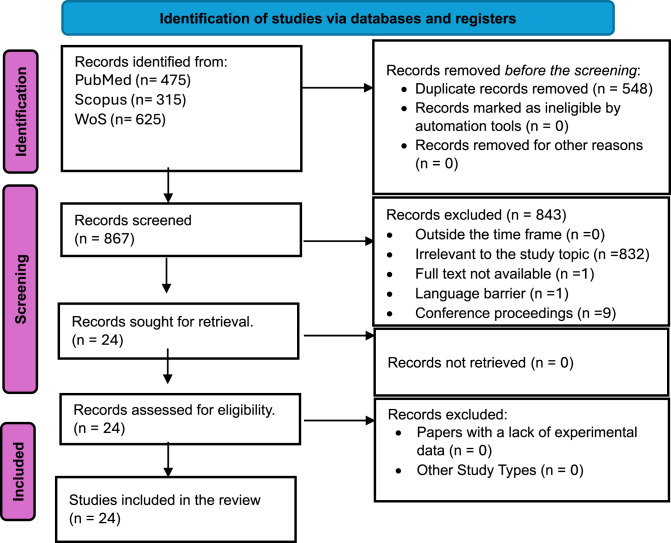
PRISMA 2020 flow diagram of the study retrieval process

A comprehensive search of three different databases yielded 1,415 items. Initially, after deleting duplicates, 867 items were retained. The titles and abstracts of these studies were then assessed, with 24 articles moving forward to the next stage. In the last stage, the studies were thoroughly reviewed to confirm that all 24 studies met the inclusion criteria of the SR. Figure 1 is a PRISMA flowchart illustrating the process for study retrieval and selection and the outcomes.

#### Geographical distribution of the studies

**Fig. 2:**
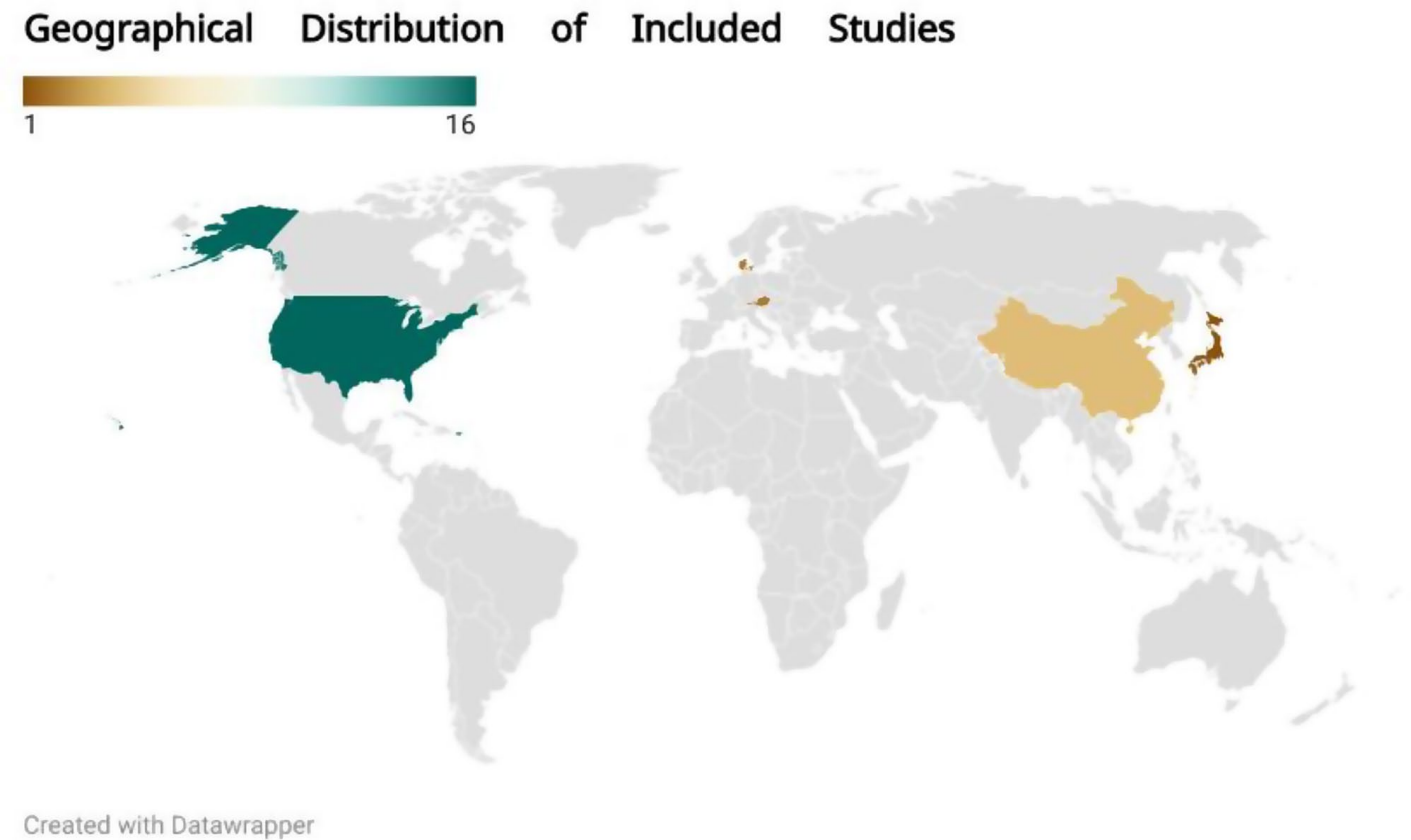
Geographical distribution of included studies

The global map in Fig. 2 shows the distribution of studies included in this review across different countries. 16 studies are from the United States, Four from China, two from Austria, one from Denmark, and one from Japan. This geographical representation highlights countries leading advancements in blood transfusion research.

#### Quality assessment

Based on the assessment conducted using the QUADAS-AI tool and according to Table 1, the risk of bias and applicability concerns of 24 studies in four domains, including patient selection, index test, reference standard, and flow and timing, were determined as follows.

**Table 1:** Detailed risk of bias and applicability concerns per study

The first author (reference)	Risk of Bias				Applicability Concerns		
	Patient Selection	Index Test	Reference Standard	Flow and Timing	Patient Selection	Index Test	Reference Standard
Ngufor, C. [19]	●	●	●	●	●	●	●
Ngufor, C. [20]	●	●	●	●	●	●	●
Nguyen, M. [21]	●	●	●	●	●	●	●
N. H. Roubinian. [22]	●	●	●	●	●	●	●
Bright, R. A. [23]	●	●	●	●	●	●	●
P. Bruun-Rasmussen. [24]	●	●	●	●	●	●	●
Whitaker, B [25]	●	●	●	●	●	●	●
Wu, K. [26]	●	●	●	●	●	●	●
Yamada, C. [27]	●	●	●	●	●	●	●
Zhu, S. [28]	●	●	●	●	●	●	●
Tschoellitsch, T. [29]	●	●	●	●	●	●	●
Melnyk, V. [30]	●	●	●	●	●	●	●
Sanaiha, Y. [31]	●	●	●	●	●	●	●
Stephens, L. D. [32]	●	●	●	●	●	●	●
Wang, M. [33]	●	●	●	●	●	●	●
Trutschl, M. [34]	●	●	●	●	●	●	●
Baucom, M. R. [35]	●	●	●	●	●	●	●
Fung, MK. [9]	●	●	●	●	●	●	●
Luo, Z. [36]	●	●	●	●	●	●	●
Zhang, H. [37]	●	●	●	●	●	●	●
Kamio, T. [38]	●	●	●	●	●	●	●
Okumura, K. [39]	●	●	●	●	●	●	●
Tschoellitsch, T. [40]	●	●	●	●	●	●	●
Portela, G. T. [41]	●	●	●	●	●	●	●

●High, ●Unclear, ●Low

#### Risk of bias

- In the *Patient Selection* domain, two studies were rated as high risk and one as unclear, primarily due to insufficient details regarding the criteria or methods used for participant selection.
- In the *Index Test* domain, two studies were rated as high risk and three as unclear, mainly due to the absence of external validation, inadequate description of test execution, and limited transparency in algorithm development and validation.
- In the *Reference Standard* domain, one study was rated as high risk and two as unclear, owing to an undefined or insufficiently described reference standard.
- In the *Flow and Timing* domain, most studies were rated as low risk, with only three rated as unclear due to a lack of information on the time interval between the index test and the reference standard.

#### Applicability concerns

- In the *Patient Selection* domain, four studies were rated as high concern, driven by small sample sizes, limited representativeness of the target population, and incomplete demographic information.
- In the *Index Test* domain, five studies were rated as high concern, primarily due to uncertainty regarding the applicability of the model in real-world clinical settings.
- In the *Reference Standard* domain, three studies were rated as high concern and two as unclear, due to ambiguity or lack of appropriateness of the reference standard in the intended clinical context.

The relative distribution of risk of bias and applicability concerns based on the QUADAS-AI assessment was examined across 24 studies. Regarding risk of bias, in the *Patient Selection* domain, two studies (8.34%) had a high risk of bias, one study (4.16%) was rated as unclear, and 21 studies (87.5%) had a low risk. In the *Index Test* domain, two studies (8.34%) were judged to have a high risk, three studies (12.5%) were unclear, and 19 studies (79.16%) were rated as low risk. In the *Reference Standard* domain, one study (4.16%) had a high risk, two studies (8.34%) were unclear, and 21 studies (87.5%) had a low risk. In the *Flow and Timing* domain, the majority of studies (21 studies, 87.5%) were rated as low risk, while three studies (12.5%) were unclear; no studies were found to have a high risk in this domain.

**Fig. 3:**
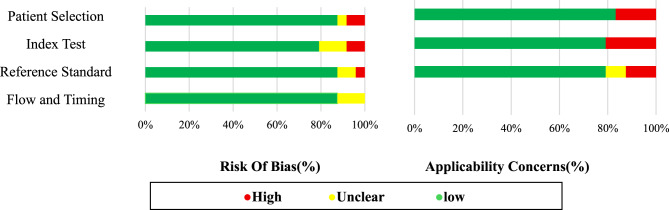
Summary of risk of bias and applicability concerns by QUADAS-AI domains

Concerning applicability concerns, high concerns in *Patient Selection* were reported in four studies (16.66%), in the *Index Test* domain in five studies (20.83%), and in the *Reference Standard* domain in three studies (12.5%). In addition, two studies (8.33%) had unclear applicability concerns in the *Reference Standard*. Details of the scoring for each study are provided in Table 1, and a summary of percentages is presented in Fig. 3

#### Clinical units and population groups studied

Based on Table 2, the majority of studies, totaling 16, were conducted on the adult population, particularly in the fields of surgery (Three studies), intensive care unit (ICU) (Three studies), trauma (two studies), cardiology (Three studies), and transplantation (two studies). The pediatric group was examined in only two studies, specifically within Hematology and neonatal care. For the geriatric population, just one study was reported in the field of nephrology. Additionally, five studies were conducted without specifying a population group (Not Applicable), N/A(), primarily related to Hematology.

**Table 2:** Clinical units and population groups studied

Population Group/clinical units	Surgery	ICU	Trauma	Pulmonology	Cardiology	Hematology	Neonatal	Transplantation	Nephrology	N/A
Adult	**3**	**3**	**2**	**1**	**3**	**1**	**0**	**2**	**0**	**1**
Pediatric	**0**	**0**	**0**	**0**	**0**	**1**	**1**	**0**	**0**	**0**
Geriatric	**0**	**0**	**0**	**0**	**0**	**0**	**0**	**0**	**1**	**0**
N/A	**0**	**0**	**0**	**0**	**0**	**3**	**0**	**0**	**0**	**2**

### Classification of AI models

In some studies, multiple AI models were utilized, and the model with the best performance is reported in Table 3. According to this table, the AI models used for detecting and forecasting ATRs are categorized into four main groups. The first category is dedicated to regression-based and statistical models, which include algorithms such as Least Absolute Shrinkage and Selection Operator (Lasso) Regression, Elastic Net Regression (ENR), logistic regression, Cox hazard regression model, linear marginal structural model, Targeted Maximum Likelihood Estimation (TMLE), and the Shakespeare Method. A total of 11 studies utilized these models. The second category comprises binary classification and tree-based machine learning models, which involve algorithms like Random Forest (RF), AdaBoost, Extreme Gradient Boosting (XGBoost), and Classification and Regression Tree (CART), with a frequency of nine studies. The third category includes deep learning and computer vision methods, where two studies employed Convolutional Neural Network (CNN) and You Only Look Once (YOLO) models for ATRs identification and prediction. Finally, the fourth category pertains to large language models (LLMs), which includes the use of chatbots and Chat Generative Pretrained Transformer (ChatGPT) in two studies.

**Table 3:** AI models by classification and clinical applications

Study ID	The first author (reference)	AI model(s)	classification of AI models	Model performance metrics and value	Data type	Type of complications	Clinical units
1	C. Ngufor [19]	AdaBoost	Binary classification and Tree-based Machine Learning Models	AUROC = 0.868, Accuracy = 0.792, Sensitivity = 0.734, Specificity = 0.824	text	Hemorrhage	Surgery
2	C. Ngufor [20]	RF	Binary classification and Tree-based Machine Learning Models	AUROC = 0.84, Sensitivity = 0.78	text	Hemorrhage	ICU
3	M. Nguyen [21]	TMLE	Regression-based and Statistical Models	RR (mortality at 24 hours = 0.89 , hemostasis at 24 hours = 1.07 hemostasis in the HH group compared to the LL group = 2.49, hemostasis in the LH group compared to the LL group = 1.94)	text	Mortality	Trauma
4	N. H. Roubinian [22]	CART	Binary classification and Tree-based Machine Learning Models	AUROC = 0.83, Sensitivity = 0.63, Specificity = 0.85	text	Pulmonary transfusion reactions	Pulmonology
5	R. A. Bright [23]	The Shakespeare Method	Regression-based and Statistical Models	Proportion difference (heart PTAEs = 0.47; *p* = 0.02, lung PTAEs = 0.37; *p* = 0.049)	text	Morbidity	Cardiology
6	P. Bruun-Rasmussen [24]	TMLE	Regression-based and Statistical Models	ATEs (Male donors vs. natural course female = 0.64, male = 1.83) Female donors vs. natural course (female = 0.62, male = −0.23) Male donors vs. female donors (female = 0.02, male = 2.06))	text	Mortality	Hematology
7	B. Whitaker [25]	binary classification algorithms	Binary classification and Tree-based Machine Learning Models	threshold = 0.9: (Sensitivity = 67.9, Specificity = 97.5, PPV = 84, AUROC = 0.92)	text	ARs	Hematology
8	K. Wu [26]	CNN	Deep Learning and Computer Vision	Accuracy = 99.8, Precision = 0.975, Sensitivity = 0.0991, F1-score = 0.983	image	IARI	Hematology
9	C. Yamada [42]	Lasso Regression	Regression-based and Statistical Models	Risk thresholds (tV-12/Kg of 30 ml/Kg, tV-12/eTBV of 30%, and RBC unit age of 7.95 days).	text	Hyperkalemia	Hematology
10	S. Zhu [28]	XGBoost	Binary classification and Tree-based Machine Learning Models	0.71AUROC=	text	Hyperbilirubinemia	Neonatal
11	T. Tschoellitsch [29]	RF	Binary classification and Tree-based Machine Learning Models	AUROC = 0.810, NPV = 0.987 PPV = 0.110, F1-score = 0.150	text	Adverse cardiovascular events	Surgery
12	V. Melnyk [30]	ENR	Regression-based and Statistical Models	Sensitivity = 0.80,Specificity = 0.69, Accuracy = 0.765	text	Morbidity	Transplantation
13	Y. Sanaiha [31]	RF	Binary classification and Tree-based Machine Learning Models	AUROC = 0.850	text	Mortality	Cardiology
14	L. D. Stephens [32]	Chat Bots	Large Language Models	N/A	text	TA-GVHD	Hematology
15	M. Wang [33]	Logistic Regression	Regression-based and Statistical Models	OR (presence of comorbidities of coagulopathy = 1.36, transplant = 1.99)	text	TACO	N/A
16	M. Trutschl [34]	YOLO algorithm	Deep Learning and Computer Vision	F1- score = 0.96, Precision = 0.942, Sensitivity = 0.977	image	AHTR	Hematology
17	M. R. Baucom [35]	Logistic regression	Regression-based and Statistical Models	OR ( pRBC transfusion volume = 1.09, FFP transfusion volume = 1.08, FFP/pRBC ratio = 1.18)	text	Mortality	Trauma
18	M. K. Fung [9]	ChatGPT	Large Language Models	N/A	text	N/A	N/A
19	Z. Luo [36]	logistic regression	Regression-based and Statistical Models	Univariate OR (age = 1.03, Bleeding complications = 3.11, FFP transfusion = 1.07) Multivariate OR (age = 1.06, Bleeding complications = 4.23, FFP transfusion = 1.09)	text	Mortality	ICU
20	H. Zhang [37]	logistic regression	Regression-based and Statistical Models	OR = 1.73	text	Infection	Surgery
21	T. Kamio [38]	RF	Binary classification and Tree-based Machine Learning Models	AUROC = 0.705, Sensitivity = 0.784, F1-score = 0.596 Precision = 0.492, Accuracy = 0.657	text	Hemorrhage	ICU
22	K. Okumura [39]	Cox hazard regression model	Regression-based and Statistical Models	transfusion HR (One-y Mortality = 1.07, One-y graft failure = 1.09)	text	Infection	Transplantation
23	T. Tschoellitsch [40]	RF	Binary classification and Tree-based Machine Learning Models	AUROC = 0.73, NPV = 0.90 PPV = 0.32, Sensitivity = 067, Accuracy = 0.67, F1-score = 0.43	text	AKI	Nephrology
24	G. T. Portela [41]	linear marginal structural model	Regression-based and Statistical Models	Risk difference (30-day MACE = 15.2)	text	Adverse cardiovascular events	Cardiology

Based on the data from Table 3, various performance evaluation metrics have been utilized across multiple studies, indicating the diversity of methods used to assess the performance of AI models in classifying and anticipating ATRs. The metrics Area Under the Receiver Operating Characteristic Curve (AUROC) and sensitivity were the most frequently used, appearing in nine studies. Following them, accuracy and F1-score were employed in five studies each. Specificity and odds ratio (OR) were reported in four studies each. The metrics Positive-Predictive Value (PPV) and precision appeared in three studies, while Negative-Predictive Value (NPV) was used in two studies. Other metrics, such as Risk ratio (RR), proportion difference, average treatment effects (ATEs), Risk thresholds, hazard ratio (HR), and Risk difference, were each utilized in only one study.

Based on the findings, the values of key performance metrics used to evaluate the models in the studies varied significantly. The AUROC values ranged from 0.71 to 0.92. Sensitivity values varied between 0.63 and 0.977. Accuracy ranged from 0.657 to 99.8%, while specificity values ranged from 0.69 to 97.5%. F1-scores ranged from 0.43 to 0.983. PPV values varied from 0.110 to 84%, and precision values ranged from 0.492 to 0.975.

Additionally, NPV values were reported as 0.987 and 0.90. Other specific metrics, such as RR, proportion difference, ATEs, OR, HR, and risk difference, were used to assess particular aspects of model performance. Studies indicate that AI models used text data as input in 22 studies, while only two studies employed CNN and the YOLO model to analyze image data.

#### Applications of AI models in clinical settings

Based on the findings in Table 3, various AI models have been applied for the identification, prediction, and classification of different outcomes associated with ATRs. For examining the relationship between blood transfusion and bleeding outcome, AdaBoost and RF models have been used. To explore the association between mortality outcomes and blood transfusion, models such as TMLE, logistic regression, and RF have been employed. Additionally, models including the Shakespeare method and ENR have been applied to investigate the effect of blood transfusion on morbidity and clinical complications. Logistic regression and Cox proportional hazards regression models have also been used to evaluate the relationship between blood transfusion and infectious complications. Binary classification models have been used to identify allergic reactions (ARs) in electronic health records (EHRs). To evaluate the effect of blood transfusion on transfusion-related pulmonary reactions, a CART model was applied, and a CNN model was used for automatic classification of incomplete antibody reaction intensity (IARI). Moreover, models such as Lasso regression and XGBoost were employed to identify factors associated with the incidence of hyperbilirubinemia and transfusion-associated hyperkalemia (TAH).

RF and Marginal Structural Models have been utilized to study the effect of blood transfusion on cardiovascular complications. Chatbots have been applied for the classification of transfusion-associated graft-versus-host disease (TA-GVHD), and the YOLO model has been used to predict acute hemolytic transfusion reactions (AHTR). Logistic regression has also been used to evaluate the prevalence of TACO and its associated risk factors. Finally, the RF model has been employed to predict acute kidney injury (AKI) in the context of individual transfusions in specific patients.

The studies conducted have focused on nine different clinical units. The highest number of studies is dedicated to Hematology, with six studies. Following that, the surgical, ICU, and cardiovascular departments each have three studies in subsequent ranks. Organ transplantation and hospital trauma each have two studies. Additionally, nephrology, pulmonology, and neonatology each have one study. In two studies, the therapeutic department is unspecified.

The studies have addressed various types of Complications, with the highest number of studies focusing on mortality (Five studies). Hemorrhage (Three studies) has also received significant attention. Cardiovascular complications, morbidity and infections each have two studies. At the same time, other events under investigation include pulmonary reactions post-transfusion, ARs, IARI, hyperkalemia, hyperbilirubinemia, AKI, TA-GVHD, TACO, and acute hemolytic reactions, each of which has one study. In one study, the type of complications was N/A.

Among the 24 reviewed studies, none explicitly reported the development, implementation, or clinical evaluation of an active AI-based management system. The studies were primarily limited to identifying, predicting, and classifying ATRs.

## Discussion

### Principal findings

The results of this SR showed that in the 24 included studies, the AI models employed for detecting and forecasting ATRs were classified into four categories: Binary Classification and Tree-based Machine Learning Models, Regression-based and Statistical Models, Deep Learning and Computer Vision, and LLMs. Among the employed models within the four main categories, RF had the highest frequency of use, being applied in five studies. Logistic regression models were used in four studies, the TMLE model was used in two studies, while other models were reported in only one study. In the included studies, the most essential model evaluation metrics were AUROC and Sensitivity, each reported in nine studies, followed by Accuracy and F1-Score, each reported in five studies.

Moreover, AI models have primarily been applied in four main focal areas: risks and outcomes of blood transfusion, risk and moderating factors, volume and intensity of transfusion, and classification and extraction of ATRs. Despite the increasing number of studies on the identification and prediction of ATRs using AI methods, this review did not find any research reporting the development, implementation, or evaluation of an AI-based active management system. Regarding the clinical focus, most studies addressed transfusion-related mortality (five studies) and Hemorrhage (three studies). Cardiovascular complications, infections, and morbidity were each investigated in two studies. Single studies were dedicated to transfusion-related pulmonary reactions, acute kidney injury, ARs, IARI, hypokalemia, hyperbilirubinemia, TACO, TA-GVHD, and acute hemolytic transfusion reactions. From the perspective of clinical units, the highest number of studies was conducted in Hematology (six studies), followed by cardiology, surgical, and the ICU units, each with three studies.

### Clinical implications of blood transfusion and AI applications

Based on the main findings of the included studies, the application of AI in clinical Implications of blood transfusion has been organized into main focal areas, as presented in Table 4.

**Table 4:** Main focal areas of the study

Main focal	Key findings	Reference	Implications
Risks and outcomes of blood transfusion	Preoperative plasma transfusion increases the risk of bleeding and related complications.	C. Ngufor [19]	Blood transfusion is a life-saving but high-risk intervention; safe and restrictive transfusion strategies should be implemented.
	Bleeding in patients receiving plasma transfusion was associated with prolonged ICU length of stay.	C. Ngufor [20]	
	More accurate predictions of adverse events during blood exchange in neonates	S. Zhu [28]	
	Perioperative blood transfusion was associated with increased 30-day mortality.	Sanaiha, Y.[31]	
Risk and moderating factors	Pre-transfusion NT-proBNP levels demonstrated good capability in distinguishing TACO.	N. H. Roubinian [22]	Careful evaluation of patient characteristics, donor factors, and biomarkers is vital for informed transfusion decision-making.
	Circulating blood volume, hemoglobin level, and body weight are among the most critical predictors of bleeding complications in patients undergoing Extracorporeal Membrane Oxygenation (ECMO) therapy.	T. Kamio [38]	
	A history of coagulation disorders and organ transplantation increases the risk of TACO.	M. Wang [33]	
	Younger blood recipients and those with brain death were associated with an increased risk of graft rejection and 1-year mortality.	K. Okumura [39]	
	Various factors were associated with the occurrence of AKI during blood transfusion episodes.	T. Tschoellitsch [40]	
	Identification of transfusion-associated cardiac complications.	R. A. Bright [23]	
	Assessment of the likelihood of massive allogeneic blood transfusion during surgery using all available features at admission.	T. Tschoellitsch [29]	
	Using red blood cell units exclusively from sex-matched donors improves patient survival compared to the current practice.	P. Bruun-Rasmussen [24]	
	A liberal transfusion strategy, compared to a restrictive one, provides benefits for patients with acute MI and anemia without the need for individual patient matching.	G. T. Portela [41]	
Volume and intensity of transfusion	A 1:1:1 transfusion ratio was associated with reduced mortality, while higher plasma-to-PRBC and platelet-to-PRBC ratios increased the likelihood of faster hemostasis.	M. Nguyen [21]	Monitoring and adjusting the transfusion volume according to key patient characteristics is a crucial factor in reducing adverse events.
	Increased volumes of RBCs, platelets, plasma, and cryoprecipitate were associated with a higher risk of transfusion-related complications.	V. Melnyk [30]	
	High transfusion volume was associated with an increased in-hospital mortality.	M. R. Baucom [35]	
	Total transfused blood volume was a determinant of hyperkalemia in children.	C. Yamada [42]	
	Each additional unit of blood increased the risk of surgical site infection by 27%.	H. Zhang [37]	
	Increased Fresh Frozen Plasma (FFP) volume in ECMO patients was associated with higher mortality.	Z. Luo [36]	
Classification and extraction of ATRs	Chatbot responses to British Society for Haematology (BSH) guidelines represent a potential tool for clinical guidance.	L. D. Stephens [32]	AI models, such as NLP, Chatbots, and ChatGPT, can serve as a key tool for extracting and classifying events from recorded data in repositories. But greater caution is required when applying their results.
	Natural Language Processing(NLP) can be utilized for extracting unstructured medical data and monitoring adverse events.	B. Whitaker [25]	
	A deep learning algorithm for the identification and classification of monocytes, relevant to risk assessment and bleeding-related complications.	M. Trutschl [34]	
	Deep learning demonstrated higher accuracy and efficiency in classifying the severity of incomplete antibody reactions.	K. Wu [26]	
	AI outperformed human experts in the identification of TACO and TRALI.	M. K. Fung [9]

1. Risks and outcomes of blood transfusion
  The reviewed studies demonstrated that transfusion of plasma and blood products may itself result in serious complications, including increased bleeding, infections, renal failure, and even higher mortality. In transplant patients, transfusion was associated with an elevated risk of graft rejection and reduced survival. This finding highlights a paradox: while blood and its components are regarded as life-saving interventions, they can simultaneously impose a considerable burden of risk. Such evidence underscores the necessity of implementing restrictive strategies and data-driven, AI-assisted decision-making approaches [43].

2. Risk and moderating factors
  Patient-specific characteristics such as a history of coagulation disorders, preoperative hematocrit levels, or donor-recipient status play a decisive role in the occurrence of transfusion-related complications. For example, donor-recipient sex matching and biomarker levels such as N-terminal prohormone brain natriuretic peptide (NT-proBNP) were shown to be significant predictors of adverse outcomes. These findings suggest that transfusion decisions should not rely solely on general guidelines but rather be tailored to the individual patient’s profile and specific risk factors. This is precisely where personalized medicine can contribute, by integrating and analyzing multiple variables to optimize donor-recipient matching, thereby reducing ATRs and enhancing transfusion safety [44].

3. Volume and intensity of transfusion
  The volume of transfused blood and blood products has been identified as one of the most critical predictors of complications, such as hyperkalemia in pediatric patients, increased mortality, or prolonged hospitalization. This finding underscores the significant association between the amount of transfusion and the severity of transfusion-related adverse outcomes. Accordingly, precise monitoring of transfusion volume and the application of intelligent tools to determine safe thresholds are of vital importance [45].

4. Classification and extraction of ATRs
  AI models such as ChatGPT have demonstrated the ability to extract unstructured medical data and monitor ATRs. Additionally, models like CNN and YOLO have shown superior performance compared to specialists in classifying monocytes and categorizing the severity of incomplete antibody reactions. The use of chatbots has also provided practical support for responding to clinical transfusion guidelines. However, the utilization of AI-generated information in transfusion medicine, such as that offered by ChatGPT, raises concerns regarding the potential for errors and variability in responses [46]. Therefore, greater caution should be exercised when utilizing the results derived from these models.

### Studies comparison and analysis

In this study, various criteria were used to evaluate models with a wide range of value domains. The wide range of values reflects the performance fluctuations of models and their dependency on data, architecture, input data types, and training parameters. Four criteria that have been most commonly used in studies include AUROC, sensitivity, accuracy, and F1-score, which indicate the focus of research on the model’s ability to distinguish between positive and negative cases and accurately identify actual instances according to the model’s objective. The AUROC criterion has been employed in all studies within the Binary classification and Tree-based Machine Learning Models category for model evaluation. AUROC is one of the most commonly used metrics for assessing binary classification performance, representing the probability that a randomly chosen positive sample will have a higher score than a randomly chosen negative sample [47].

In studies where AUROC values were reported, some models showed better performance than others. For example, Whitaker et al. [25], using binary classification models to extract features from EHRs, achieved the highest AUROC of 0.92. This study assessed only 605 out of 86,618 blood transfusion cases without reactions, and this imbalance limited the generalizability of the calculated PPV on the test set to the entire population. Therefore, AUROC inherently has limitations, especially in imbalanced datasets, which can lead to overly optimistic results [3]. Negofor et al. [19], employing multiple models to investigate the effect of pre-surgical plasma transfusion on bleeding, obtained the highest AUROC of 0.868 with the AdaBoost model, indicating strong model performance.

Among studies that used the RF model, Negofor et al. [20] reported an AUROC of 0.84, and Sannai et al. [31] reported an AUROC of 0.85. Studies have shown that RF is a powerful model in machine learning that is widely used for classification and prediction, particularly performing well on imbalanced datasets, primarily when the importance of features is evaluated based on AUROC. RF shows superior performance in ranking and selecting relevant variables compared to other methods [48, 49].

Zhou et al. [28] evaluated several models and reported that XGBoost had the highest AUROC of 0.71 among the models, though this value was suboptimal due to limitations in the training set. To address this limitation, Xv et al. introduced the AUROC-mixup approach, where synthetic (mixup) data is used in deep AUROC optimization, enhancing the model’s generalization ability on small datasets, particularly in imbalanced medical tasks [50]. Relying solely on the AUROC value in the test set provides a limited view of the model’s generalization ability [51]. Therefore, alongside this criterion, other criteria of the confusion matrix [52] have also been utilized.

Among the studies, precision, sensitivity, and F1-score were used to evaluate the YOLO model in the Trotzel study [34] and the CNN model in the Wu study [26]. In the Trotzel study, the values for these metrics were 0.942, 0.977, and 0.96, respectively, while in the Wu study, they were 0.975, 0.991, and 0.983, respectively. These metric values are the highest among the studies in comparison. The common feature of these two studies is that the input data to the models are images. The results of the Trotzel study showed that the CNN model outperformed three immunologists in classifying IARI automatically. In the Wu study, the YOLO model classified and identified monocytes in smear images faster and more accurately than manual classification. A review of these two studies reveals that image-based models such as YOLO and CNN play a significant role in reducing diagnostic time and improving the accuracy of hematology specialists [53, 54]. This is precisely what the metric values substantiate. Overall, the diversity in evaluation criteria and data types underscores the importance of selecting appropriate evaluation metrics and aligning them with the data type and clinical application. Directly comparing model performance without considering the context and data volume used may be misleading.

A study by Melnyk et al. [30] employed the Elastic Net regression model to investigate the association between blood product transfusion and the occurrence of complications or short-term mortality following lung transplantation. The model achieved an accuracy of approximately 76.5%; the study sample size (369 patients) was relatively limited. To address scenarios with small sample sizes, Chen et al. proposed a transfer learning-based approach for high-dimensional stochastic frontier models incorporating Elastic Net, which demonstrated improved estimation accuracy [55].

In another study conducted by Stephens et al. [32], aimed at comparing and evaluating the recommendations provided by several chatbots regarding the irradiation of blood products to prevent TA-GVHD, it was found that chatbots inconsistently suggested inaccurate indications for TA-GVHD prevention. Although chatbots have the potential to provide helpful support in delivering health information, significant concerns remain regarding their safety and reliability [56]. When selecting an AI model, factors such as efficiency, flexibility, scalability, and ease of implementation are critical [57]. However, beyond the results of model evaluation, careful consideration should be given to sample size, data quality, the representativeness of input samples, appropriate feature selection, and potential limitations. Addressing these aspects can substantially enhance the reliability and generalizability of the model.

Several studies have investigated NIATRs. Roubinian [22] and Whitaker [25] investigated pulmonary and ARs, which are categorized as acute immune-mediated adverse reactions. Yamada [42], Wang [33], and Trutschl [34] examined reactions such as hyperkalemia, TACO, and AHTR, classified as acute non-immune-mediated adverse reactions. Additionally, Stephens [32] studied transfusion-associated TA-GVHD, which represents a delayed immune-mediated reaction. Acute reactions typically occur within 24 hours, while delayed reactions may arise up to 48 hours after transfusion. Therefore, future efforts should focus on more accurately identifying patients at risk for acute reactions and providing blood products with improved compatibility to minimize adverse outcomes [58]. AI models possess a high capacity for classifying and anticipating NIATR-type reactions [59].

High bias in the domains of Patient Selection and Index Test can limit the generalizability of study results. Suppose the patient population in the real world differs from the patients on whom the training and/or validation data of a medical AI model were based (for example, the use of small samples, non-representative populations, or a lack of demographic information). In that case, the performance of the AI model may be compromised, and this performance degradation can occur unevenly across different patient groups [60]. Additionally, models trained in a specific setting and exhibiting high performance in predicting ATRs may perform poorly in other settings. This phenomenon, known as “sampling bias” [61], can lead to unfair and harmful decision-making in real clinical environments To mitigate sampling bias, advanced statistical methods are being developed that aim to address bias by identifying the target population [62].

Additionally, in the Index Test domain, the absence of external validation, lack of transparency in describing test implementation methods, or insufficient details regarding algorithm development and training can lead to overfitting and reduced model performance in real-world settings. Consequently, this diminishes the accuracy estimates and lowers clinical trust in the model. Evaluation frameworks such as QUADAS-AI recommend addressing these weaknesses and reporting methods transparently to enable the development of models applicable across multiple clinical environments [63].

Our review highlights a vital research gap regarding vulnerable populations, particularly pediatric patients. Examination of the population groups included in the reviewed studies indicates that research has been predominantly focused on the adult population, whereas studies involving Pediatric and Geriatric remain limited. According to emerging evidence [64–66] on age-dependent differences in physiological responses, AI models for predicting ATRs must incorporate pediatric-specific variables and perform stratified evaluations to ensure fairness and safety across all age groups. Therefore, the design and validation of tailored and optimized models for high-risk groups represent a critical next step in AI research in the field of hemovigilance.

### Application of AI for detecting ATRs in clinical units

Although AI models have been applied across various clinical units, such as Hematology, Surgery, ICU, Cardiology, Trauma, and Transplantation, units with higher transfusion rates and consequently greater risk of ATRs have received less attention. For instance, one study reported that the highest proportion of transfusions within a hospital, 39.9%, occurred in the obstetrics and gynecology department [67]. Another study demonstrated that transfusions were more frequent in emergency departments compared to other units [68]. Therefore, expanding the application of AI models in these high-risk settings is essential to leverage better their potential for predicting complications and reducing ATRs.

The expansion of AI model applications in clinical settings requires obtaining necessary approvals to ensure compliance with safety, quality, and ethical standards. Despite the greater focus of most studies on the development of AI models, few have examined their translation into commercially available tools usable at the bedside [69, 70]. For the practical use of AI models, their risk level must be assessed and approved by regulatory authorities such as the United States Food and Drug Administration (U.S. FDA). According to the classification by the U.S. FDA, medical devices, including AI-based tools, are categorized into three classes: Class I, indicating low risk; Class II, indicating moderate to relatively high risk; and Class III, indicating high risk [71]. Since ATRs pose significant dangers to patient health, the use of AI models in this domain is considered to carry a relatively high risk. Therefore, the implementation of preventive measures and the establishment of appropriate hemovigilance systems are essential [72, 73], and before market introduction, necessary FDA approvals for premarket approval (PMA) must be obtained.

The European Union AI Act (EU AI Act) also classifies AI applications in healthcare as “high-risk,” subjecting them to stringent requirements regarding transparency, safety, and privacy. [74] The ultimate goal of predictive models is to enhance patient safety and reduce transfusion risks; accordingly, under the EU AI Act, clinical deployment of these models is conditional upon guaranteeing patient safety, ensuring that the model itself does not increase patient risk.

The International Society of Blood Transfusion (ISBT) also holds a stringent perspective on integrating AI with hemovigilance monitoring. This society emphasizes addressing the need for educational resources, standards, and regulatory frameworks supported by AI models [75].

### AI in the management of ATRs

Based on the findings, the number of studies applying AI models to identify and predict adverse transfusion events has gradually increased between 2015 and 2025. Only two studies were published in 2015 and 2016, but from 2020 onwards, the number of studies rose steadily, reaching six publications in 2022. In 2023, six additional studies were published, and ultimately, seven studies were conducted in 2024. Despite research advancements, this review did not identify any study on the development or evaluation of AI-based active management systems for ATRs. Clinical Decision Support Systems (CDSSs) can be deployed at the POC to assist in managing ATRs [76]. Although previous studies have demonstrated that appropriately designed CDSSs can enhance transfusion safety, most of these systems have been rule-based or heuristic, and no reports are available on fully automated AI-based CDSSs for the active management of ATRs [77–79].

This highlights a gap between model development and its practical clinical application for ATRs management [10]. Despite the promising capabilities of AI in improving the safety and efficiency of blood transfusion, further research is essential to bridge the gap between model development and its clinical application [80]. The implementation of AI models should not only ensure high accuracy and effectiveness but also practical usability, reproducibility, and adaptability to real-world conditions. A study [81] utilized the Surgical-Personalized Anticipation of Transfusion Hazard (S-PATH) model to predict the risk of blood transfusion during surgery, based on data from over 3 million surgical procedures across 45 hospitals in the United States. Results demonstrated that the S-PATH algorithm outperformed the conventional maximum surgical blood ordering schedule (MSBOS) method in predicting transfusion needs more accurately and efficiently, while also reducing unnecessary blood type test orders. This algorithm can serve as a valuable tool in preoperative clinical decision-making in similar settings.

Other studies [82, 83] employing advanced information retrieval techniques, such as NLP on EHR, have shown that physician reporting is often incomplete, and automated systems can outperform manual reporting in terms of response time and sensitivity. Although numerous studies have examined the application of AI models in laboratory environments, only a small percentage of research in the field of ATRs has undergone prospective or external validation. This highlights the critical need to evaluate the performance of these models under real-world conditions before widespread clinical deployment.

### Strengths

The strengths of this study are notable in several aspects. First, a rigorous search strategy using standardized keywords was employed to ensure that all relevant studies were identified and included. To assess the quality of the studies, the QUADAS-AI tool was utilized, which is specifically designed for evaluating AI-related research and enables transparent reporting of risk of bias and applicability concerns based on predefined criteria. This SR examined the diversity of AI models, data types, clinical settings, and various aspects of ATRs. Its multifaceted approach enhanced the comprehensiveness and reliability of the study, making it highly relevant for both clinicians and researchers. By thoroughly analyzing previous research, this review provided a clear and up-to-date overview of how AI methods are applied to identify and predict ATRs. Moreover, the findings laid a foundation for the development and improvement of AI models and opened new avenues for future research. From a health policy perspective, the results can inform strategies to prevent ATRs and promote the use of innovative electronic systems over traditional methods. In the field of medical informatics, these findings provide a novel perspective on AI applications, enabling researchers to identify gaps better and leverage them in future studies.

### Challenges

Despite its high potential for predicting ATRs, AI faces multiple challenges. One of the significant issues is the lack of integration of AI models with existing EHRs. AI algorithms require complete and standardized data to function accurately, whereas transfusion-related data in EHRs are often fragmented, incomplete, or stored in inconsistent formats. Another critical challenge concerns accountability in AI-assisted decision-making [84]. When AI guides clinical decisions regarding transfusions, it remains unclear who is responsible in the event of an error: the physician, the model developer, or the healthcare institution?. This ambiguity can undermine clinicians’ trust and hinder widespread adoption of AI systems, highlighting the need for clear legal and ethical frameworks to define responsibilities and accountability.

According to FDA requirements for medical AI systems, any clinical use of algorithms necessitates undergoing a thorough evaluation process of safety, efficacy, and performance transparency, which is both time-consuming and costly [85]. The use of AI in hemovigilance processes may necessitate changes to existing workflows and staff training, which could temporarily reduce operational efficiency. Implementation and maintenance costs of these systems represent another considerable challenge, particularly for hospitals and centers with limited resources. Additionally, bias present in AI models can perpetuate discrimination and inequality, often stemming from biased data, algorithmic unfairness, and lack of diversity in AI development [86].

From a technical perspective, the development of necessary infrastructures for model implementation is essential. To harmonize transfusion-related datasets and address the challenges of heterogeneity in EHR, interoperability standards such as Health Level 7/Fast Healthcare Interoperability Resources (HL7/FHIR) can be utilized [87]. The HL7 FHIR standard represents a significant advancement in healthcare information exchange, addressing longstanding interoperability challenges in healthcare systems [19]. Practical applications of this standard include improving data sharing in healthcare, particularly transfusion-related data, among hospitals and medical centers, which has been demonstrated in health systems integration projects [20].

One of the essential steps in the process of bias mitigation is model interpretability, as it enables understanding how decisions are generated [60, 88]. This capability is not only crucial for building user trust and comprehension but also essential for complying with legal requirements and ensuring the ethical deployment of AI [89]. In situations where algorithmic biases reduce model performance and exacerbate inequalities, interpretability frameworks such as SHapley Additive exPlanations (SHAP) and Local Interpretable Model-agnostic Explanations (LIME) can help clarify the decision-making process, thereby facilitating model acceptance and gaining clinicians’ trust [90, 91]. To overcome these barriers, strategies include improving stakeholder engagement, providing adequate training, and addressing legal and ethical concerns [92]. Understanding and addressing these implementation barriers is crucial for healthcare leaders and policymakers to effectively incorporate AI technologies, ultimately benefiting patients and clinical staff [93].

### Limitations and future directions

This study provides valuable insights but is not without limitations. One limitation was the lack of access to the full text of some articles; despite extensive database searches and contacting authors, some vital information may have been missed. Additionally, relevant studies that were not indexed in the searched databases may have been overlooked. Another limitation is that only articles published in English were included, potentially excluding necessary research from other leading countries. To enhance the accuracy and comprehensiveness of future studies, it is recommended to expand the scope of databases searched and to include studies published in multiple languages.

One of the significant limitations of the current studies is the absence of research on the implementation or clinical evaluation of AI-based active management systems for ATRs. Despite the availability of promising predictive models, evidence regarding their impact after integration into clinical settings and on the management of clinical outcomes is lacking. This research gap imposes a direct limitation on our ability to conclude the translational potential of these models into clinical practice. Another limitation of this study is that the literature search was restricted to the past ten years. Although AI has witnessed remarkable growth during this period, valuable studies outside this timeframe may have enriched the findings and better supported the study objectives. Therefore, it is recommended that future research consider a broader time range when conducting literature searches. Another critical point is that AI models and tools can be categorized based on the timing of clinical transfusion events into three stages: before transfusion for the prevention of ATRs, during transfusion for the active management of ATRs, and after transfusion for treatment and the promotion of patient health. It is recommended that researchers consider and apply this framework in future studies.

During the evaluation of studies using the QUADAS-AI tool, several studies were found to have a high or unclear risk of bias in patient selection, index test, reference standard, and flow and timing. as well as a high or unclear risk in patient selection, index test, and reference standard in terms of applicability (particularly the studies by Fung [9] and Whitaker [25]). This indicates limitations in the generalizability and real-world applicability of the models, which affects the robustness of their results. Future research should focus on addressing these limitations to achieve higher-quality models.

## Conclusion

The findings of this study indicate that AI models in the 24 reviewed studies were primarily applied across four Main focal areas: predicting the risks and outcomes of blood transfusion, identifying risk and modulating factors, examining the relationship between transfusion volume and intensity, and classifying and extracting ATRs. Within each domain, a variety of models were utilized, including Regression-based and Statistical Models, Binary classification and Tree-based Machine Learning Models, Deep Learning and Computer Vision, and large language models for predicting ATRs. To enhance the generalizability and reliability of these models, regardless of their evaluation outcomes, careful consideration must be given to sample size, data quality, accurate representation of input samples, proper feature selection, and potential limitations.

Although AI models demonstrate considerable potential for identifying and predicting ATRs, the present review revealed a critical gap between evidence and clinical application, as no study reporting the implementation or clinical evaluation of AI-based active management systems was identified. This highlights the gap between model development and its practical clinical application for ATRs management. Therefore, future studies should prioritize external validation, clinical impact trials, real-world integration of models with EHRs, cost-benefit analyses, and transparent reporting by Prediction model Risk Of Bias Assessment Tool (PROBAST), Transparent Reporting of a Multivariable Prediction Model for Individual Prognosis or Diagnosis (TRIPOD), and Consolidated Standards of Reporting Trials-Artificial Intelligence (CONSORT-AI) guidelines.

The present review identified an additional research gap concerning vulnerable populations, particularly pediatric patients. Analysis of the studied demographic groups indicated that the majority of research has focused on adults, whereas studies involving Pediatric and Geriatric populations remain limited. To predict ATRs, AI models must incorporate pediatric-specific variables and perform stratified evaluations to ensure fairness and safety across all age groups.

Based on the findings, Individual patient factors and transfusion volume play a pivotal role in the occurrence of complications. Therefore, implementing safe transfusion strategies, including the use of CDSS integrated with EHRs and personalized medicine approaches, is essential in future research. Adherence to ethical considerations and the protection of patient privacy are also of paramount importance in the use of these tools.

## Electronic supplementary material

Below is the link to the electronic supplementary material.

Supplementary Material 1

## Data Availability

The authors have stated that all data provided in this article are available for sharing.

## Abbreviations

ATRs: Adverse Transfusion Reactions
NIATRs: Non-infectious ATRS
AI: Artificial Intelligence
POC: Point of Care
TACO: Transfusion-Associated Circulatory Overload
TRALI: Transfusion-Related Acute Lung Injury
PROSPERO: International Prospective Register of Systematic Reviews
SR: Systematic Review
PRISMA: Preferred Reporting Items for Systematic Reviews and Meta-Analyses
MeSH: Medical Subject Headings
QUADAS-AI: Quality assessment of diagnostic accuracy studies-artificial intelligence
ICU: Intensive Care Unit
N/A: Not Applicable
LASSO: Least Absolute Shrinkage and Selection Operator
ENR: Elastic Net Regression
TMLE: Targeted Maximum Likelihood Estimation
RF: Random Forest
XGBoost: Extreme Gradient Boosting
CART: Classification And Regression Trees
CNN: Convolutional Neural Network
YOLO: You Only Look Once
LLMs: Large Language Models
ChatGPT: Chat Generative Pretrained Transformer
AUROC: Area Under the Receiver Operating Characteristic Curve
OR: Odds Ratio
PPV: Positive Predictive Value
NPV: Negative-Predictive Value
RR: Risk Ratio
ATEs: Average Treatment Effects
HR: Hazard Ratio
ARs: Allergic Reactions
EHRs: Electronic Health Records
IARI: Incomplete Antibody Reaction Intensity
TAH: Transfusion-Associated Hyperkalemia
TA-GVHD: Transfusion-Associated Graft-Versus-Host Disease
AHTR: Acute Hemolytic Transfusion Reactions
AKI: Acute Kidney Injury
MACE: Major Adverse Cardiovascular Events
pRBC: Packed Red Blood Cell
FFP: Fresh Frozen Plasma
BSH: British Society for Hematology
NT-proBNP: N-Terminal Prohormone Brain Natriuretic Peptide
ECMO: Extracorporeal Membrane Oxygenation
NLP: Natural Language Processing
ADRs: Adverse Drug Reactions
U.S. FDA: United States Food and Drug Administration
PMA: Premarket Approval
EU AI Act: The European Union AI Act
ISBT: International Society of Blood Transfusion
CDSS: Clinical Decision Support System
S-PATH: Surgical-Personalized Anticipation of Transfusion Hazard
MSBOS: Maximum Surgical Blood Ordering Schedule
HL7/FHIR: Health Level 7/Fast Healthcare Interoperability Resources
SHAP: Shapley Additive Explanations
LIME: Local Interpretable Model-Agnostic Explanations
PROBAST: Prediction Model Risk of Bias Assessment Tool
TRIPOD: Transparent Reporting of a Multivariable Prediction Model for Individual Prognosis or Diagnosis
CONSORT-AI: Consolidated Standards of Reporting Trials-Artificial Intelligence

## Declarations

### Registration and protocol

This study was conducted according to a protocol registered in PROSPERO under the identifier CRD420251019252, which is accessible at https://www.crd.york.ac.uk/PROSPERO/view/CRD420251019252.
