# Supplementary material for: Artificial intelligence in the identification and prediction of adverse transfusion reactions(ATRs) and implications for clinical management: a systematic review of models and applications
Source: BMC Med Inform Decis Mak. 2025 Oct 28;25:396. doi: 10.1186/s12911-025-03232-z (PMC12560389; doi:10.1186/s12911-025-03232-z)
Supplement: Supplementary file 1 — Supplementary Material 1 [file 12911_2025_3232_MOESM1_ESM.docx]

**Table A1.** Search strategy

| **Database** | **Search strategy** |
| --- | --- |
| PubMed | (("Artificial Intelligence"[MeSH Terms] OR "Artificial Intelligence"[Title/Abstract] OR "AI"[Title/Abstract] OR "Machine Learning"[MeSH Terms] OR "machine learning*"[Title/Abstract] OR "Natural Language Processing"[MeSH Terms] OR "neural networks, computer"[MeSH Terms] OR "neural network*"[Title/Abstract] OR "Expert Systems"[MeSH Terms] OR "expert system*"[Title/Abstract] OR "decision support systems, clinical"[MeSH Terms] OR "clinical decision support system*"[Title/Abstract] OR "CDSS"[Title/Abstract] OR "Data Mining"[Title/Abstract] OR "intelligent system*"[Title/Abstract]) AND ("Blood Transfusion"[MeSH Terms] OR "blood transfusion*"[Title/Abstract] OR "Platelet Transfusion"[Title/Abstract] OR "Plasma Transfusion"[Title/Abstract] OR "RBC Transfusion"[Title/Abstract] OR "transfusion*"[Title/Abstract]) AND ("Transfusion Reaction"[MeSH Terms] OR "transfusion reaction*"[Title/Abstract] OR "allergic reaction*"[Title/Abstract] OR "TRALI"[Title/Abstract] OR " Hemolytic Transfusion Reaction"[Title/Abstract] OR " Transfusion Associated Graft Vs. Host Disease"[Title/Abstract] OR "Transfusion-Related Acute Lung Injury"[Title/Abstract] OR "Adverse Transfusion Reaction*"[Title/Abstract] OR "Adverse Effect*"[Title/Abstract] OR "Side Effect*"[Title/Abstract] OR "Safety"[Title/Abstract] OR "Blood Safety"[MeSH Terms] OR "Risk*"[Title/Abstract] OR "Hazard*"[Title/Abstract] OR "Complication*"[Title/Abstract] OR "Adverse Event*"[Title/Abstract])) AND (2015/1/1:2025/8/6[pdat]) |
| Scopus | ( TITLE-ABS ( "Artificial Intelligence" OR "AI" OR "Machine Learning" OR "Natural Language Processing" OR "neural network" OR "neural networks" OR "Expert System" OR "clinical decision support system" OR "CDSS" OR "Deep learning" OR "Data Mining" OR "intelligent system" ) AND ( TITLE-ABS ( "Blood Transfusion" OR "Blood Transfusions" OR "Platelet Transfusion" OR "Plasma Transfusion" OR "RBC Transfusion" OR "transfusion" ) ) AND ( TITLE-ABS ( "Transfusion Reaction" OR "Transfusion Reactions" OR "allergic reaction" OR "allergic reactions" OR "TRALI" OR "adverse transfusion reaction" OR "adverse transfusion reactions" OR "Adverse Event" OR "adverse effect" OR "Adverse Reaction" OR "Adverse Reactions" OR "Blood Transfusion Reaction" OR "side effect" OR "Safety" OR "Blood Safety" OR "risk" OR "hazard" OR "complication") ) ) AND PUBYEAR > 2014 AND PUBYEAR < 2026 |
| WoS | (TS= ( "Artificial Intelligence" OR "AI" OR "Machine Learning" OR "Natural Language Processing" OR "neural network" OR "neural networks" OR "Expert System" OR "clinical decision support system" OR "CDSS" OR "Deep learning" OR "Data Mining" OR "intelligent system" ) AND (TS= ( "Blood Transfusion" OR "Blood Transfusions" OR "Platelet Transfusion" OR "Plasma Transfusion" OR "RBC Transfusion" OR "transfusion" ) ) AND (TS= ( "Transfusion Reaction" OR "Transfusion Reactions" OR "allergic reaction" OR "allergic reactions" OR " TRALI " OR " Transfusion-Related Acute Lung Injury" OR "adverse transfusion reaction" OR "adverse transfusion reactions" OR "Adverse Event" OR "adverse effect" OR "Adverse Reaction" OR "Adverse Reactions" OR "Blood Transfusion Reaction" OR "side effect" OR "Safety" OR "Blood Safety" OR "risk" OR "hazard" OR "complication") ) ) AND PY=(2015 OR 2016 OR 2017 OR 2018 OR 2019 OR 2020 OR 2021 OR 2022 OR 2023 OR 2024 OR 2025) |

**Table A2.** Reasons for Exclusion of Studies Not Meeting Inclusion Criteria

| Number of Articles | Reason for Exclusion |
| --- | --- |
| 832 | Irrelevant to the study topic |
| 1 | Language barrier |
| 9 | Conference proceedings |
| 1 | Full text not available |

**Table A3.** Results Of the Data Extraction Process

| **Study ID** | **The first author (reference)** | **Year** | **Type of Study** | **country** | **Aim of study** | **Key findings** | **Type of Complications** | **population group (pediatric/adult/geriatric)** | **Clinical Units** | **Model Performance Metrics and**  **Value** | **AI model(s)** | **Data Type for AI Model** |
| --- | --- | --- | --- | --- | --- | --- | --- | --- | --- | --- | --- | --- |
| 1 | Ngufor, C. (1) | 2015 | observational cohort study | USA | The aim of this study is to utilize advanced machine learning methods to investigate the relationship between preoperative plasma transfusion (in patients with elevated International Normalized Ratio [INR]) and perioperative Hemorrhage. | The results demonstrated that preoperative plasma transfusion (PPT) may increase the risk of Hemorrhage and related complications. Furthermore, machine learning methods showed higher accuracy in predicting Hemorrhage compared to traditional statistical approaches, with boosting algorithms performing particularly better. | Hemorrhage | adult | surgery | AUROC=0.868  Accuracy =0.792  PCC.se=0.017  AUC.se=0.016  Sensitivity =0.734  Sensitivity.se=0.031  Specificity=0.824  Specificity.se=0.020 | AdaBoost | text |
| 2 | Ngufor, C. (2) | 2016 | Retrospectivestudy | USA | The aim of this study is to examine the impact of Hemorrhage on prolonged ICU length of stay (ICU-LOS) in patients receiving plasma transfusion (PPT) and to identify patient subgroups with differential responses using a causal and predictive framework. | The findings of this study show that Hemorrhage in patients receiving plasma transfusion leads to a longer stay in the intensive care unit (ICU). Specifically, Hemorrhage significantly increases the risk of prolonged ICU stay. However, if the patient does not experience Hemorrhage, their ICU length of stay is slightly reduced. | Hemorrhage | adult | intensive care unit (ICU) | AUROC=0.84  Sensitivity=0.78  geometric mean =0.75 | RF | text |
| 3 | Nguyen, M. (3) | 2020 | secondary data analysis | USA | The aim of this study is to examine the dynamic impact of transfusion ratios (platelets, plasma, and red blood cells) on the outcomes of severely injured patients. | In the intention-to-treat analysis, the 1:1:1 transfusion ratio was associated with a reduction in mortality, although this was not statistically significant. For patients whose hemostasis took longer than 2 hours, the 1:1:1 ratio significantly increased the probability of achieving hemostasis starting from the 4th hour. In the per-protocol analysis of actual transfusion ratios received, no significant association was found between transfusion ratios and mortality across four consecutive time intervals. However, patients receiving both high plasma:PRBC and high platelet:PRBC ratios had a 2.49-fold increased relative risk (95% CI: 1.19–5.22) of achieving hemostasis during the 3rd hour after admission, indicating a significant beneficial effect of higher plasma and platelet transfusion ratios on hemostasis. | mortality | adult | trauma | Risk ratio (RR) of mortality at 24 hours= 0.89  RR of hemostasis at 24 hours= 1.07  RR of hemostasis in the HH group compared to the LL group= 2.49  RR of hemostasis in the LH group compared to the LL group= 1.94 | TMLE | text |
| 4 | N. H. Roubinian, D.(4) | 2020 | case  control study | USA | The main objective of the study is to investigate the utility of NT-proBNP levels in assisting the diagnosis and differentiation of various types of pulmonary transfusion reactions. | Pre-transfusion NT-proBNP levels were significantly higher in patients with ARDS and TACO. NT-proBNP levels were higher in patients with sepsis accompanied by ARDS or TRALI. NT-proBNP showed good ability to distinguish TACO from controls (AUC=0.83), similar to echocardiography. | pulmonary transfusion reactions | adult | Pulmonology | AUROC=0.83  Sensitivity =0.63  Specificity =0.85 | CART | text |
| 5 | Bright, R. A. (5) | 2021 | Retrospective analytical-methodological study | USA | objective was to develop a method of using EHR notes to  find recognized and unrecognized potential TAEs (PTAEs), which incidentally might also uncover other anomalies. We wanted our method to operate in the setting of the above-noted challenges. | The Shakespeare method successfully identified PTAEs. The three top-scoring documents in cardiovascular topics topic 17, heart valve repair; topic 33, tapped pericardial effusion; topic35, coronary artery bypass graft; topic 42, heart attack; and topic 11, vascular repair) were associated with cardiovascular PTAEs:  atrial fibrillation, tachycardia, bradycardia, other heart rhythm  abnormality, or hypotension, which are features of TAEs | Morbidity | adult | Cardiology | heart PTAEs (Proportion difference = 0.47; P = 0.02)  lung PTAEs ((Proportion difference=0.37; P=.049)) | The Shakespeare Method | text |
| 6 | P. Bruun-Rasmussen. (6) | 2022 | retrospective cohort study, | Denmark | The aim of this study is to investigate the effect of red blood cell (RBC) donor sex on the mortality of transfused recipients. | The results obtained from emulating trials using targeted learning suggest that treating male patients with RBC units exclusively from male donors increases the 28-day survival compared with the current practice. Further, transfusing female patients with RBC units exclusively from donors of either sex increases patient survival compared with the current practice where patients can receive a mix of female and male donated RBC units. | mortality | adult | Hematology | Average treatment effects (ATEs)  (  Male donors vs. natural course (female=.64, male=1.83)  Female donors vs. natural course (female=.62, male=-0.23)  Male donors vs. female donors (female=.02, male=2.06)) | TMLE | text |
| 7 | B. Whitaker. (7) | 2022 | retrospective -analytical study | USA | The aim of this study is to identify and predict adverse reactions related to blood transfusion using both structured and unstructured data from electronic health records (EHRs) and to develop machine learning models based on natural language processing (NLP) feature extraction. | The application of NLP techniques to extract information from unstructured data in biomedical sources, including EHRs, has the potential to improve both surveillance and research. all models performed similarly. The validation set metrics matched the performance of the CV estimates with an AUC of 0.92. This was not significantly different from that of the CV sets of 0.91 ± 0.03 (with a confidence interval of one standard deviation). | Allergic reactions | N/A | Hematology | threshold = 0.9: (Sensitivity = 67.9, Specificity = 97.5, positive predictive  value (PPV^+^) = 84)  AUROC=0.92 | binary classification algorithms (logistic regression, decision tree, random forest, k-nearest  neighbors, and naïve Bayes) | text |
| 8 | Wu, K. (8) | 2022 |  | China | In this study, our aim was to develop a novel deep learning model for automatic classification of incomplete antibody reaction intensity (IARI) that is robust against the effects of imbalanced sample distribution among IARI categories and interference from artifacts in the HCM-HDMS Coombs test. | This study presents a fast and fully automatic deep learning model for classifying the intensity of incomplete antibody reactions, which performs more accurately and efficiently than previous models and even surpasses the performance of immunologists. | incomplete antibody reaction intensity (IARI) | N/A | Hematology | Accuracy=99.8  Precision=0.975  Sensitivity  =.0991  F1-score=0.983 | CNN | image |
| 9 | Yamada, C. (9) | 2022 | Case-Control | USA | The aim of this study is to identify factors associated with the occurrence of transfusion-associated hyperkalemia (TAH) in pediatric patients using multivariate analyses and artificial intelligence techniques. | The total blood transfusion volume within 12 hours (tV-12) per kilogram of body weight was the most significant factor associated with the occurrence of transfusion-associated hyperkalemia (TAH) in children. | Hyperkalemia | Pediatric | Hematology | Risk thresholds  ) tV-12/Kg of 30 ml/Kg, tV-12/eTBV of 30%, and  RBC unit age of 7.95 days). | Lasso Regression | text |
| 10 | Zhu, S. (10) | 2022 | retrospective study | China | Identification of risk factors associated with adverse events in neonates with hyperbilirubinemia who underwent exchange transfusion (ET) within the first 30 days of birth. | This study demonstrated that explainable artificial intelligence (XAI) provides more accurate predictions of adverse events during exchange transfusion in neonates and helps clinicians better understand the nonlinear relationships between clinical factors. | hyperbilirubinemia | Pediatric | Neonatal | 0.71AUROC= | XGBoost | text |
| 11 | Tschoellitsch, T. (11) | 2022 | retrospective cohort study | Austria | Objective Is it possible to predict massive perioperative  allogeneic blood transfusion using machine learning? | In this study, out of 3782 patients, 139 received at least 10 units of pRBC. Using all the available features at the time of admission, the Random Forests model was able to accurately predict the likelihood of massive perioperative allogeneic blood transfusion. The best result was achieved with an AUC of 0.810 and a high NPV of 0.99. | adverse cardiovascular events | adult | surgery | AUROC=0.810  NPV=0.987  PPV=0.110  F1-score= 0.150 | RF | text |
| 12 | Melnyk, V. (12) | 2023 | Retrospective study | USA | This study aims to use machine learning to determine the association between transfusions of blood products and short-term morbidity and mortality after lung transplantation. | Increasing volumes of packed red blood cells, platelets, plasma, and cryoprecipitate transfused during the operation and within 72 hours postoperatively were associated with a higher risk of complications. Additionally, preoperative functional dependence, preoperative blood transfusion, use of ECMO, and antifibrinolytic therapy increased the risk of complications. In contrast, preoperative steroid use, taller stature, and primary chest closure were protective factors. | Morbidity | Adult | transplantation | Sensitivity=0.80  Specificity =0.69  Accuracy =0.765 | Elastic Net Regression (ENR) | text |
| 13 | Sanaiha, Y.(13) | 2023 | retrospective cohort study | USA | The aim of the study was to examine the association between perioperative transfusion and 30-day mortality in cardiac surgery patients. | The main findings showed that higher preoperative hematocrit levels were associated with reduced mortality, renal failure, and prolonged mechanical ventilation, while perioperative transfusions were linked to increased 30-day mortality. Additionally, machine learning models identified transfusions as the most important predictor of mortality, demonstrating a direct dose-response relationship between transfusions and adverse outcomes. the study authors created 3 unique models for the prediction of 30-day mortality, each adjusted for baseline patient and surgical characteristics, in addition to either total transfusions, RBC transfusions, or CP transfusions. The area under the curves for these 3 models were 0.850, 0.830, and 0.814, respectively. | mortality | adult | Cardiology | AUROC=0.850 | RF | text |
| 14 | Stephens, L. D. (14) | 2023 | comparative analysis | USA | Evaluation and comparison of indication lists provided by multiple artificial intelligence programs (chatbots) regarding the irradiation of blood components for the prevention of transfusion-associated graft-versus-host disease (TA-GVHD), in accordance with the 2020 British Society for Hematology (BSH) practice guidelines. | The most important finding was that although the chatbot responses varied, they were all relevant, relatively complete, and generally concordant with the BSH guidelines. However, they missed some important indications listed in the guidelines and showed discrepancies in cases such as irradiation indications for fetuses and neonates. Additionally, some chatbots included errors in their lists of indications, which could lead to medical misinformation. | transfusion-associated graft-versus-host disease (TA-GVHD) | N/A | Hematology | N/A | Chat Bots | text |
| 15 | Wang, M. (15) | 2023 | retrospective case–control study | USA | This study aimed to provide a new assessment of prevalence of TACO and risks associated with development of TACO in patient populations enriched with high-risk patients, using a now nearly ubiquitous source of clinical data: electronic health records (EHR). | In this study, among 56,208 patients who received blood transfusions, only 102 (0.2%) developed TACO. A history of coagulopathy and organ transplant was associated with an increased likelihood of developing TACO. | Transfusion-associated circulatory overload (TACO) | adult | N/A | Odds Ratio (presence  of comorbidities of coagulopathy=1.36, transplant= 1.99) | Logistic Regression | text |
| 16 | Trutschl, M. (16) | 2023 | Applied Experimental Study | USA | The aim of this study is to automate the detection and classification of monocytes in microscopic images using the YOLO model to increase accuracy and speed in the evaluation of blood transfusion candidates and to reduce the risk of acute hemolytic transfusion reactions. | We developed a deep learning algorithm for detecting, localizing, and classifying monocytes. The best prediction on the training data was achieved at epoch 189. The model detected 273 monocytes in a set of 22 validation images. | Acute hemolytic transfusion reaction (AHTR) | N/A | Hematology | F1- score=0.96  Precision=0.942  Sensitivity =0.977  mAP@0.5= 0.989  mAP@.  5:.95= 0.841 | YOLO algorithm | Image |
| 17 | Baucom, M. R. (17) | 2024 | retrospective cohort study | USA | The aim of the study is to investigate whether increasing the volume of blood transfusion (packed red blood cells and fresh frozen plasma) in patients with isolated severe traumatic brain injury (sTBI) is associated with increased in-hospital mortality. | The main findings showed that in patients with isolated severe traumatic brain injury, an increased volume of blood transfusion (including packed red blood cells and fresh frozen plasma) was associated with a higher in-hospital mortality rate. In the larger dataset, mortality increased with each unit of blood transfused, reaching up to 74% in patients who received 11 or more units. | mortality | adult | trauma | Odds Ratio (  pRBC transfusion volume=1.09, FFP transfusion volume=1.08, FFP/pRBC ratio=1.18) | Logistic regression | text |
| 18 | Fung, MK. (18) | 2024 | comparative study | USA | This study aimed to evaluate ChatGPT’s ability to classify transfusion reactions, assess severity, and determine imputability using NHSN criteria, comparing its performance with an expert panel, transfusion medicine specialists, and their prior use of the TrDDx algorithm in challenging cases. | Comparison with the assessments of the transfusion medicine expert panel from the 2014 validation study showed that the AI system was accurate in less than half of the cases, which was significantly lower than the 72.1% accuracy achieved by transfusion medicine specialists on the same cases. A more detailed analysis of the AI’s accuracy revealed considerable variability across different NHSN categories; for instance, the system correctly classified all cases of TACO and TRALI, outperforming the specialists in these categories. | All reactions | N/A | N/A | N/A | ChatGPT | text |
| 19 | Luo, Z. (19) | 2024 | retrospective study | China | The aim of this study was to investigate the relationship between fresh frozen plasma (FFP) transfusion and mortality in patients receiving ECMO support, as well as to evaluate the risk factors associated with FFP transfusion. | In 116 ECMO patients, the in-hospital mortality rate was 32.8%. The amount of FFP transfused was higher in deceased patients compared to survivors (5.07 vs. 2.16 mL/kg/day, p = 0.007). After adjusting for confounding factors, FFP transfusion was significantly associated with mortality (OR = 1.09, p = 0.035). Additionally, higher APTT, elevated uric acid levels, and lower platelet counts were significant risk factors for FFP transfusion. | mortality | adult | intensive care unit (ICU) | Univariate OR (age=1.03, Bleeding complications=3.11, FFP transfusion=1.07)  Multivariate OR (age=1.06, Bleeding complications=4.23, FFP transfusion=1.09) | logistic regression | text |
| 20 | Zhang, H. (20) | 2024 | retrospective study | China | To determine whether perioperative blood transfusion increased the risk of SSI and to further explored whether there was a dose-response relationship. | In this study, the average time to diagnosis of surgical site infection after surgery was 20.5 days. The risk of infection increased by 27% with each additional unit of blood transfusion, and the risk rose significantly after more than 3 units of transfused blood. | infection | adult | surgery | OR=1.73 | logistic regression | text |
| 21 | Kamio, T. (21) | 2024 | Retrospective cohort study | Japan | The aim of the study is to predict Hemorrhage complications in patients undergoing extracorporeal membrane oxygenation (ECMO) using machine learning algorithms. | This study involved 470 patients admitted to the intensive care unit who were treated with extracorporeal membrane oxygenation (ECMO). Of these, 357 patients were included in the final analysis after excluding those who did not meet the criteria for Hemorrhage prediction. Models were developed using 47 variables to predict Hemorrhage. Among these models, the LightGBM and random forest models demonstrated the best performance, with accuracy and recall of 70.5% and 0.784, respectively. These models also showed good performance with an area under the ROC curve above 0.7. | Hemorrhage | adult | intensive care unit (ICU) | AUROC = 0.705  PR AUC=0.562  Sensitivity = 0.784  F1- score=0.596  Precision= 0.492  Accuracy= 0.657 | RF | text |
| 22 | Okumura, K. (22) | 2024 | Retrospective cohort study | USA | The aim of this study was to evaluate the association of donor blood transfusion with outcomes of liver transplantation (LT) | The findings showed that donors who received blood transfusions were younger and had a higher proportion of brain death. This group was associated with an increased risk of graft rejection, 1-year mortality, and graft failure. Additionally, blood transfusion was linked to an increased risk of graft rejection at 6 months and 1-year post-liver transplantation. | infection | adult | transplantation | transfusion hazard ratio (HR)  (One-y Mortality = 1.07, One-y graft failure= 1.09) | cox hazard regression model | text |
| 23 | Tschoellitsch, T. (23) | 2024 | retrospective cohort study. | Austria | This study aims to determine whether renal function deterioration in the context of individual transfusions in individual patients can be predicted using machine learning. Recipient and donor  characteristics linked to increased risk are identified. | In this study, acute kidney injury (AKI) occurred in 17.4% of transfusion episodes. The AKI prediction model had an AUC-ROC of 0.73, with negative and positive predictive values of 0.90 and 0.32, respectively. The results showed that recipient characteristics were more influential than donor characteristics in predicting post-transfusion AKI. | acute kidney injury (AKI) | geriatric | Nephrology | AUROC=0.73  NPV^-^ = 0.90  PPV^+^=0.32  Sensitivity =067  Area Under the Precision-Recall Curve) AUC-PR (=0.34  Accuracy =0.67  Balanced Accuracy) bACC(= 0.67  F1-score=0.43  average precision (AP)= 0.34 | RF | text |
| 24 | Portela, G. T. (24) | 2025 | Randomized Controlled Trial - RCT | USA | The aim is to use machine earning methods to determine, based on each patient’s baseline characteristics, which transfusion strategy places them at lower risk of 30-day cardiovascular complications. | This exploratory analysis of the MINT study showed that patients' baseline characteristics could not predict the effect of restrictive versus liberal transfusion strategies on 30-day mortality or 30-day death or myocardial infarction (MI). Therefore, it seems appropriate to apply the MINT study’s average treatment effect findings, which favor a liberal transfusion strategy, to all patients with acute MI and anemia represented in the MINT sample. | adverse cardiovascular events | adult | Cardiology | Risk difference (30-day MACE = 15.2( | linear marginal structural model | text |

**Table A4.** QUADAS-AI Quality Assessment

| **Risk of bias** | | | | | | | | | | | | | | | |
| --- | --- | --- | --- | --- | --- | --- | --- | --- | --- | --- | --- | --- | --- | --- | --- |
| The first author (reference) | **Patient Selection** | | | | **Index Test (AI Model)** | | | | **Reference Standard** | | | | **Flow and Timing** | | |
|  | **Answer (Yes/No/Unclear)** | **Notes/Evidence from Study** | | **Risk of Bias (Low/High/Unclear)** | **Answer (Yes/No/Unclear)** | **Notes/Evidence from Study** | | **Risk of Bias (Low/High/Unclear)** | **Answer (Yes/No/Unclear)** | **Notes/Evidence from Study** | | **Risk of Bias (Low/High/Unclear)** | **Answer (Yes/No/Unclear)** | **Notes/Evidence from Study** | **Risk of Bias (Low/High/Unclear)** |
| Ngufor, C. (1) | yes | Between January 1, 2008, and December 31, 2011, a total of 155,492 patients aged ≥ 18 years underwent noncardiac surgery at the participating institution. | | low | yes | The goal of this work is to apply advanced machine learning methods to study the relationship between preoperative plasma transfusion (PPT) and PB in patients with elevated INR undergoing  noncardiac surgery | | low | Unclear | - | | Unclear | yes | Of them, 14,743 had an INR measured within 30 days of the index surgical procedure, with 1,234 having an INR ≥ 1.5. | Low |
| Ngufor, C. (2) | yes | To be considered for study participation, patients must meet the  following criteria: age ≥ 18 years, noncardiac surgery and an INR ≥ 1.5 in the 30 days preceding surgery | | low | yes | the framework integrates a classification model for risks prediction and  a regression model to predict actual LOS | | low | yes | To demonstrate the effectiveness of the two-level approach in identifying patients at risk of longer ICU-LOS attributable to Hemorrhage as a complication of PPT, the second level regression model is compared with a regression model trained on the complete training data. | | low | yes | In this study, we first aim to identify the relationship between the ICU-LOS (length of stay in intensive care unit) and blood loss due to pre-operative plasma transfusion (PPT). Data were collected from the hospital records between 2008 and 2011. | Low |
| Nguyen, M. (3) | yes | The PROPPR study was a pragmatic, phase 3, multicenter, randomized clinical trial of 680 severely injured patients who arrived at one of 12 level I trauma centers in the US and in Canada between August 2012 and December 2013. | | low | yes | *Patients were randomized into two groups to receive one of two blood product transfusion ratios* | | low | yes | In PROPPR, anatomic hemostasis in the operating room was an objective assessment by the surgeon indicating that Hemorrhage within the surgical field was controlled and no further hemostatic interventions were anticipated. | | low | yes | Time scale in this study started at admittance into the ED (t = 0). Data from patient assessments were collected at multiple time-points, including pre-ED time for baseline variables | Low |
| N. H. Roubinian, D.(4) | yes | Pulmonary transfusion reactions were identified between May 2015 and July 2016 through active surveillance of all adult hospitalized patients who received a blood transfusion. | | low | yes | used Wilcoxon Rank-Sum tests to compare  NT-proBNP levels, and classification and regression tree (CART) algorithms to  produce a ranking of covariates in order of relative importance for differentiating TACO from transfused controls. | | low | yes | *Clinical diagnoses of TACO were derived from criteria used in National Healthcare Safety Network (NHSN) surveillance definitions... TRALI was defined using the National Heart Lung and Blood Institute (NHLBI) Working Group definition...* | | low | yes | *Prior to expert panel review, pre- and post-transfusion recipient blood samples were collected from the clinical laboratory when available for cases and controls... Timing of blood collection prior to and following transfusion was recorded.* |  |
| Bright, R. A. (5) | yes | Out of 58,976 hospital admissions, those under the age of 16 and those without notes were removed, leaving a total of 49,284 admissions. | | low | yes | The AI model is used to analyze electronic health record (EHR) notes and extract information using the "Shakespeare Method."  This method focuses on identifying and analyzing terms related to patient experiences and uses them to model potential unrecognized adverse events. Classification models like Naïve Bayes and Logistic Regression were used for feature selection and modeling. | | low | yes | evaluate the AI model, the reference standard involves at least two comparison groups:  A group of patients who received blood transfusion (T).  A comparison group of patients who did not receive a transfusion (C). | | low | yes | *The timing and flow of the analysis are detailed in the methods, which include preprocessing of the data, creation of n-gram vectors, feature extraction, topic modeling, and evaluation of the results. During the process, more than 41,664 features were extracted and applied to different data models.* | Low |
| P. Bruun-Rasmussen. (6) | yes | The study focused on patients aged 18 or older receiving in-hospital RBC transfusions in the Capital Region of Denmark from January 1, 2009, to April 10, 2018. | | low | yes | We used the doubly robust approach, TMLE, to estimate the risk of death 28 days after the baseline-transfusion  under each intervention | | low | Unclear | - | | Unclear | yes | The study followed patients for up to 28 days after their baseline transfusion. The study used real-world data from the Danish Capital Region Blood Bank Transfusion Database and adjusted for confounders identified through causal inference methods, including a causal directed acyclic graph (DAG). | low |
| Whitaker, B(7) | yes | In a 4-year period, all 146 reported transfusion ARs were pulled from a database of 86,764 transfusions in an academic health system, along with a random sample of 605 transfusions without reported ARs. | | low | yes | we explored whether machine learning methods, such as natural language processing (NLP), can identify and report transfusion allergic reactions (ARs)  from electronic health records (EHRs). | | Unclear | yes | Each of these events was identified in the EHR data as a clinician-ordered transfusion-related AE report for the Centers for Disease Control and Prevention (CDC) National Healthcare Safety Network Hemovigilance Module (NHSN-HM). Reports were filed if the clinician believed the AR was in the likelihood range from 'definite' to 'probable' on the immutability spectrum. | | low | yes | To detect potential ARs, the study assessed a time window from 6 h prior to 30 h following the transfusion procedure; analysis showed that all AR reports were observed within that period.  Because patients often receive back-to-back transfusions, those administered within 24 h of each other were considered the same transfusion episode.  Features are derived only when the evidence is found within the transfusion episode time window described in the 'Defining Cohorts and Episodes' section. | low |
| Wu, K. (8) | yes | In this study, data were collected from 1725 blood samples obtained from the Suzhou Blood Centre and the First Affiliated Hospital of Soochow University in China. Out of these, 97 samples that could not have their IARI category accurately determined were excluded, leaving 1628 samples (94.38%) for selection. | | low | yes | A deep learning model based on Convolutional Neural Networks (CNN) was used to classify the IARIs. Five different sub-models, including AlexNet, VGG, ResNet, Inception, and DenseNet, were employed to detect different IARI categories. | | low | yes | The IARI corresponding to the blood samples was obtained using the HCM-HDMS Coombs test, and 97 samples (5.62%) whose IARI category could not be accurately determined were excluded from the study. | | low | yes | Flow and Timing:  The data were split into two sets:   1. Training Set (80%) containing 1302 IARI samples for model development. 2. Testing Set (20%) containing 326 IARI samples for model evaluation. | Unclear |
| Yamada, C. (9) | yes | therefore, 12 patients in each age group, a total of 60 patients were investigated as a control group (non-TAH group). Hyperkalemia was defined as K+ level above institutional reference range (upper limit of  4.9–5.5 mmol/L depending on the patient’s age and facility), and TAH was defined as K+ increase >0.2mmol/L during or after the transfusion compared to K+ before the transfusion in the same patient. Minor K+ increase of ≤0.2 mmol/L was excluded as an acceptable variable. | | low | yes | Machine learning (ML) methods for dimensionality reduction were used... such as PCA, t-SNE, and UMAP.  ... data collected in this study included patient demographics, diagnosis, medical history and comorbidities, date and time of transfusion and recorded pre-transfusion K+ levels... | | low | yes | "Hyperkalemia was defined as K+ level above institutional reference range (upper limit of 4.9–5.5 mmol/L depending on the patient’s age and facility), and TAH was defined as K+ increase >0.2mmol/L during or after the transfusion compared to K+ before the transfusion in the same patient." | | low | yes | The date and approximate start and end times of transfusion of each unit were also recorded when available.  At each facility and for each age group, the control group (non-TAH group) included the first 3 eligible patients who did not experience TAH during the RBC transfusion or had normal follow-up potassium (K+) levels within 12 h after the completion of RBC transfusion(s). | low |
| Zhu, S. (10) | yes | The medical records of neonates who received exchange transfusions to treat severe hyperbilirubinemia in neonatal units at the Children’s Hospital, Zhejiang University School of Medicine over a period of six years (from January 2015 through December 2020) were reviewed retrospectively | | low | yes | In this study, the best performing model was enhanced  with an interpretation method called SHAP [15], which is a game-heretic approach for explaining the output  of any machine learning model by computing each feature for the prediction.  Several widely used machine learning methods... were used to train a machine learning model using 70% of the data and test them on the standby 30% of the data. | | low | yes | adverse events during ET were defined by the following quantitative criteria that were outside the nor-  mal range for neonates. Hyperglycemia occurred when  serum glucose was > 7.2 mmol/L, metabolic acidosis if  HCO 3 was < 18 mmol/L, hyperkalemia if serum potassium was ≥ 5.5 mmol/L, hypokalemia when serum  potassium was < 3.0 mmol/L, hypocalcemia if serum  calcium was < 0.9 mmol/L, thrombocytopenia if plate-  let count < 100 × 10 9 /L, hyponatremia if serum sodium  was < 135 mmol/L, cyanosis if SpO 2 < 90%, and top-up  transfusion if the hemoglobin reduction met the clinical  indications for transfusion. All indicators were monitored during the ET, following the clinical guidelines in  China. | | low | yes | Patient characteristics ... were collected at different time points before, during and after ET. | low |
| Tschoellitsch, T. (11) | yes | The study included data of patients who underwent cardiac surgery of any kind between January 1, 2010, and December 31, 2019, both with and without transfusions. | | low | yes | Five artificial intelligence algorithms were used to predict which patients would be in the RBC 10 group, which received more than 10 pRBC units. The AI models tested were: logistic regression, Random Forest, neural network, gradient boosting machine (GBM), and adaptive boosting (ADA). | | low | yes | The reference standard used to categorize patients into the RBC mod or RBC 10 groups was the number of pRBC units transfused during their hospital stay, specifically looking at whether the patient received fewer than or more than 10 units of pRBC. | | low | yes | The dataset was split into a training set (80% of the data) and a test set (20% of the data). The training set was used to train the AI models, and the test set was used to evaluate their performance. Data from the test set was not used for training, avoiding data leakage. | Unclear |
| Melnyk, V. (12) | yes | The project is a retrospective electronic chart review of all adults (18 years of age and above) patients  who underwent single- and double-lung transplantation at our tertiary center within a 5-year period between January 1, 2013, and December 31, 2017. | | low | yes | Following imputation, Elastic Net Regression (ENR) was performed  using the R package “eNetXplorer” (v. 1.1.1) for the sample. ENR is  an approach that helps address high number of features/variables as  well as those that may have higher multicollinearity. | | Unclear | yes | "The composite outcome included six hard endpoints: mortality during index hospitalization; primary graft dysfunction grade 3 at 72 h; the need for postoperative mechanical circulatory support; neurological complications including stroke, seizure, or encephalopathy; perioperative acute coronary syndrome (non-ST-segment elevation or ST-segment elevation myocardial infarction) or cardiac arrest; and renal dysfunction requiring renal replacement therapy." | | low | yes | The final database encompassed a broad spectrum of clinical factors of interest. The list included preoperative patient attributes, donor factors, surgical and anesthetic details of the index operation, detailed perioperative blood product transfusion history, and complications up to the point of initial hospital discharge or death before discharge. | low |
| Sanaiha, Y.(13) | yes | **Inclusion Criteria:**   - Adult patients undergoing isolated CABG, isolated valve, CABG with a single valve, and multivalve surgeries between 2013 and 2019.   **Exclusion Criteria:**   - Patients with infective endocarditis. - Preoperative Hematocrit (HCT) <20% or >45%. | | low | yes | Random Forest classification models were used to predict 30-day mortality, considering patient and surgical characteristics, along with transfusion data (total transfusions, pRBC transfusions, or CP transfusions). | | low | yes | The reference standard for the study included various clinical outcomes defined by the Society of Thoracic Surgeons (STS), such as:  30-day all-cause mortality.  Postoperative prolonged mechanical ventilation (>24 hours after surgery).  Acute renal failure (defined by an increase in serum creatinine).  Stroke. | | low | yes | Data Collection Period: Data were collected between 2013 and 2019 from the Academic Cardiac Surgery Consortium.  Data Analysis Process:   - The study used logistic regression models first to analyze the association of preoperative HCT with postoperative outcomes.   Then, Random Forest models were developed and validated using 70% of the data for training and 30% for testing. | low |
| Stephens, L. D. (14) | no | - | | High | yes | A prompt was trialed on June 15, 2023 on 4 generative AI pro-  grams (Table 1): ChatGPT-3.5, ChatGPT-4, Bard, and Bing Chat. | | low | yes | Grades for concordance and completeness vis-à-vis BSH guide-  lines were expressed as the average of the independent reviewers’  grades. | | low | yes | Responses were reviewed by all authors to determine feasibility of the project. Subsequently, the following prompt was presented on  June 27, 2023 to 4 generative AI programs: “List the indications for   - irradiation of blood components for prevention of TA-GVHD. | Unclear |
| Wang, M. (15) | yes | A total of 56,208 unique patients who received blood transfusions at least once at UCSF between January 1, 2012, and August 30, 2022, were included. Out of these, 2202 (3.9%) developed potential reactions leading to transfusion reaction investigations, resulting in 3178 transfusion reaction reports. | | low | yes | The index test in this study was the use of a custom-written, rule-based Natural Language Processing (NLP) algorithm designed to extract key features of transfusion reactions, including reaction dates, types, case definitions, severity, and imputability from unstructured transfusion reaction reports. | | low | yes | The reference standard for identifying TACO cases was the clinical judgment of transfusion specialists who reviewed transfusion reaction reports, using the case definitions, severity levels, and imputability criteria outlined by the CDC's National Healthcare Safety Network (NHSN). | | low | yes | The data collection followed a retrospective design. The study involved reviewing transfusion reaction reports collected between 2012 and August 2022. The NLP algorithm was applied to extract the relevant data within the reports, and the analysis was conducted based on the matched cohorts of TACO cases and controls. The control cohort was matched to TACO cases at a 5:1 ratio by age and sex. | low |
| Trutschl, M. (16) | yes | Data were obtained from 109 microscopic images collected at a single blood center (LifeShare Blood Center). | | High | yes | The index test was the YOLOv5s model for monocyte detection and classification. | | low | yes | Reference standard was manual annotation of monocytes with location and class labels. | | low | yes | All samples were processed after annotation.  Clear separation between training, validation, and test sets. | low |
| Baucom, M. R. (17) | yes | The study used a retrospective cohort design, utilizing data from the Trauma Quality Improvement Program (TQIP) database between 2017 and 2019. The focus was on patients diagnosed with isolated severe traumatic brain injury (sTBI) due to blunt or penetrating trauma. In total, 82,780 patients met the criteria for isolated sTBI with an Abbreviated Injury Scale (AIS) score of 3 or greater for the head region. Additionally, data was collected from a single-center trauma registry, which included 6367 patients with a diagnosis of TBI from January 1, 2013, to December 31, 2021. | | low | yes | statistical analyses, including univariate logistic regression and multiple logistic regression, were performed to identify factors associated with in-hospital mortality. | | low | no | - | | High | yes | The study analyzed patients who received pRBC transfusions within 4 hours of arrival. A secondary analysis was performed to assess the ratio of FFP to pRBC transfusions and its association with 30-day mortality | low |
| Fung, MK. (18) | no | An AI system was requested to assess 36 case scenarios to provide a diagnosis, severity, and imputability of the transfusion reactions using the  CDC National Healthcare Safety Network (NHSN) criteria. | | Unclear | yes | The 36 aforementioned transfusion reaction scenarios  were entered into one generative AI program, ChatGPT-  3.5 (OpenAI; San Francisco, CA; version May 24, 2023),  from May 29, 2023 to June 1, 2023 with the following  prompt: “Using NHSN criteria, what is the case definition  criteria, severity, and imputability for the following sus-  pected transfusion reaction: [Insert scenario]. | | High | yes | All responses from each group were compared against the 2014 expert panel's diagnostic determinations, which were considered to be the correct response. | | low | yes | The AI system's initial response to all cases was recorded and analyzed. No AI responses were regenerated to maintain consistency in analysis.  The 36 aforementioned transfusion reaction scenarios were entered into one generative AI program... from May 29, 2023 to June 1, 2023... | low |
| Luo, Z. (19) | yes | Study Population: The study involves a retrospective review of all patients requiring ECMO at the Department of Critical Care Medicine at West China Hospital of Sichuan University, from January 2014 to June 2020.  Exclusion Criteria: Age <18 years, ECMO duration <1 day, and incomplete information. | | low | yes | Boruta algorithm was used for feature ranking and selection to identify significant variables related to ECMO patients' in-hospital mortality. | | low | yes | the reference standard appears to be in-hospital mortality of ECMO patients, as the study focused on factors associated with mortality during hospitalization. | | low | yes | Study Design: A retrospective review conducted over a period from January 2014 to June 2020.  Data Collection: Data were collected from the hospital’s digital system, and statistical analyses were performed using R software. | low |
| Zhang, H. (20) | yes | The study was conducted on 124 patients with surgical site infections (SSI) following instrumented spinal deformity fusions from 2011 to 2021, and 248 "control" patients. | | low | yes | to examine the effects  of mismatched variables, we further adjusted for possible confounding factors using conditional  logistic regression models | | low | yes | - | | High | yes | Study Duration: The study was conducted over a period of 2 years, from 2011 to 2021.  Data Collection: Data was collected from patients' electronic medical records. | low |
| Kamio, T. (21) | yes | dult patients (≥18 years) who underwent emergency or unscheduled admissions from January 1, 2018, to June 30, 2022. | | low | yes | The AI model predicts Hemorrhage complications using several machine learning algorithms including:  Support Vector Machine (SVC)  Random Forest  Extreme Gradient Boosting (XGB)  Light Gradient Boosting Machine (LightGBM) | | low | yes | he definition of a positive Hemorrhage surrogate event is based on the transfusion of four or more units of RBCs within 4 hours after ECMO initiation, with a total of four or more units administered within the subsequent 24 hours. | | High | yes | The study uses data from six hospitals in the Tokushukai Medical Group, including ICU and emergency care unit (ECU) data. Data Collection Period: From January 1, 2018, to June 30, 2022.  Preprocessing: Preprocessing includes identifying outliers, imputing missing values, and standardizing continuous variables.  Dataset Splitting: The dataset is split into training (80%) and test (20%) sets. | low |
| Okumura, K. (22) | yes | Donor transfusion information was available from 2004 onward in the UNOS database, with transfusion levels categorized as: no transfusion, 1-5 units, 6-10 units, and >10 units.  Follow-up data was available until September 30, 2023. | | low | yes | Recipient survival analysis was performed by KaplaneMeier method and multivariable  Cox-hazard model. | | High | yes | Comparison between the two recipient groups: those who received organs from transfused donors and those from non-transfused donors. | | low | yes | The follow-up period for the data extends until September 30, 2023.  **Statistical Tests**:  The Kolmogorov-Smirnov test was used to evaluate the normality of continuous variables. | low |
| Tschoellitsch, T. (23) | yes | Data from patients who received a transfusion between November 1, 2016, and August 31, 2020. | | low | yes | Machine learning approach using random forests to predict posttransfusion renal dysfunction (AKI). | | low | yes | AKI is defined by an increase in creatinine from pretransfusion baseline:  Either 0.3 mg/dL or a factor of 1.5 from baseline, measured within 12 hours post-transfusion up to 7 days or until the next transfusion.  Creatinine levels are used as the basis for labeling AKI episodes. | | low | yes | Data was extracted from electronic health records, including transfusion data and laboratory results. Invalid transfusion episodes (with insufficient data) were discarded.  Data cleaning and imputation were performed to handle missing values and outliers. Features were selected based on the availability of data (less than 15% missing values). | low |
| Portela, G. T. (24) | yes | This is an open-label, randomized trial that included 3,504 patients aged 18 years or older with ST-segment elevation or non-ST-segment elevation MI (myocardial infarction) and anemia (hemoglobin <10g/dL) from 144 clinical sites across 6 countries. | | low | yes | Machine learning methods were used to identify baseline patient characteristics that informed the individualized treatment  effect of a restrictive versus liberal transfusion strategy for each patient. | | Unclear | yes | The primary outcome was the 30-day all-cause death or MI composite endpoint. | | low | yes | Participants were randomized to receive either a restrictive or liberal transfusion strategy. Patients were followed for 30 days post-randomization to assess clinical outcomes. | yes |
| **Applicability Concerns** | | | | | | | | | | | | | | | |
| The first author (reference) | **Patient Selection** | | | | **Index Test (AI Model)** | | | | **Reference Standard** | | | | - | | |
|  | **Answer (Yes/No/Unclear)** | | **Notes/Evidence from Study** | **Risk of Bias (Low/High/Unclear)** | **Answer (Yes/No/Unclear)** | | **Notes/Evidence from Study** | **Risk of Bias (Low/High/Unclear)** | **Answer (Yes/No/Unclear)** | | **Notes/Evidence from Study** | **Risk of Bias (Low/High/Unclear)** |  | | |
| Ngufor, C. (1) | yes | | - | low | yes | | - | low | no | | - | Unclear |  |  |  |
| Ngufor, C. (2) | yes | | - | low | yes | | - | low | yes | | - | low |  |  |  |
| Nguyen, M. (3) | yes | | - | low | yes | | - | low | yes | | - | low |  |  |  |
| N. H. Roubinian, D.(4) | yes | | - | low | yes | | - | low | yes | | - | low |  |  |  |
| Bright, R. A. (5) | yes | | - | low | yes | | - | low | yes | | - | low |  |  |  |
| P. Bruun-Rasmussen. (6) | yes | | - | low | yes | | - | low | Unclear | | - | Unclear |  |  |  |
| Whitaker, B(7) | yes | | - | High | yes | | - | High | yes | | - | low |  |  |  |
| Wu, K. (8) | yes | | - | low | yes | | - | low | yes | | - | low |  |  |  |
| Yamada, C. (9) | yes | | - | High | yes | | - | low | yes | | - | low |  |  |  |
| Zhu, S. (10) | yes | | - | low | yes | | - | low | yes | | - | low |  |  |  |
| Tschoellitsch, T. (11) | yes | | - | low | yes | | - | low | yes | | - | low |  |  |  |
| Melnyk, V. (12) | yes | | - | low | yes | | - | High | yes | | - | low |  |  |  |
| Sanaiha, Y.(13) | yes | |  | low | yes | | - | low | yes | | - | low |  |  |  |
| Stephens, L. D. (14) | no | | - | High | yes | | - | low | yes | | - | low |  |  |  |
| Wang, M. (15) | yes | | - | low | yes | | - | low | yes | | - | low |  |  |  |
| Trutschl, M. (16) | yes | | - | low | yes | | - | low | yes | | - | low |  |  |  |
| Baucom, M. R. (17) | yes | | - | low | yes | | - | low | no | | - | High |  |  |  |
| Fung, MK. (18) | no | | - | High | yes | | - | High | yes | | - | low |  |  |  |
| Luo, Z. (19) | yes | | - | low | yes | | - | low | yes | | - | low |  |  |  |
| Zhang, H. (20) | yes | | - | low | yes | | - | low | yes | | - | High |  |  |  |
| Kamio, T. (21) | yes | | - | low | yes | | - | low | yes | | - | High |  |  |  |
| Okumura, K. (22) | yes | |  | low | yes | | - | High | yes | | - | low |  |  |  |
| Tschoellitsch, T. (23) | yes | | - | low | yes | | - | low | yes | | - | low |  |  |  |
| Portela, G. T. (24) | yes | | - | low | yes | | - | High | yes | | - | low |  |  |  |

References:

1. Ngufor C, Murphree D, Upadhyaya S, Madde N, Kor D, Pathak J. Effects of Plasma Transfusion on Perioperative Bleeding Complications: A Machine Learning Approach. Stud Health Technol Inform. 2015;216:721-5.

2. Ngufor C, Murphree D, Upadhyaya S, Madde N, Pathak J, Carter R, et al. Predicting Prolonged Stay in the ICU Attributable to Bleeding in Patients Offered Plasma Transfusion. AMIA Annual Symposium proceedings AMIA Symposium. 2016;2016:954-63.

3. Nguyen M, Pirracchio R, Kornblith LZ, Callcut R, Fox EE, Wade CE, et al. Dynamic impact of transfusion ratios on outcomes in severely injured patients: Targeted machine learning analysis of the Pragmatic, Randomized Optimal Platelet and Plasma Ratios randomized clinical trial. Journal of Trauma and Acute Care Surgery. 2020;89(3):505-13.

4. Roubinian NH, Chowdhury D, Hendrickson JE, Triulzi DJ, Gottschall JL, Looney MR, et al. NT‐proBNP levels in the identification and classification of pulmonary transfusion reactions. Transfusion. 2020;60(11):2548-56.

5. Bright RA, Rankin SK, Dowdy K, Blok SV, Bright SJ, Palmer LAM. Finding Potential Adverse Events in the Unstructured Text of Electronic Health Care Records: Development of the Shakespeare Method. JMIRx Med. 2021;2(3):e27017.

6. Bruun-Rasmussen P, Andersen PK, Banasik K, Brunak S, Johansson PI. Estimating the effect of donor sex on red blood cell transfused patient mortality: A retrospective cohort study using a targeted learning and emulated trials-based approach. eClinicalMedicine. 2022;51.

7. Whitaker B, Pizarro J, Deady M, Williams A, Ezzeldin H, Belov A, et al. Detection of allergic transfusion-related adverse events from electronic medical records. Transfusion. 2022;62(10):2029-38.

8. Wu K, Duan S, Wang Y, Wang H, Gao X. Convolutional neural network-based automatic classification for incomplete antibody reaction intensity in solid phase anti-human globulin test image. Medical & Biological Engineering & Computing. 2022;60(4):1211-22.

9. Yamada C, Edelson MF, Lee AC, Saifee NH, Dinov ID. Transfusion-associated hyperkalemia in pediatric population: Analyses for risk factors and recommendations. Transfusion. 2022;62(12):2503-14.

10. Zhu S, Zhou L, Feng Y, Zhu J, Shu Q, Li H. Understanding the risk factors for adverse events during exchange transfusion in neonatal hyperbilirubinemia using explainable artificial intelligence. BMC Pediatr. 2022;22(1):567.

11. Tschoellitsch T, Boeck C, Mahecic TT, Hofmann A, Meier J. Machine learning-based prediction of massive perioperative allogeneic blood transfusion in cardiac surgery. European Journal of Anaesthesiology. 2022;39(9):766-73.

12. Melnyk V, Xu W, Ryan JP, Karim HT, Chan EG, Mahajan A, et al. Utilization of machine learning to model the effect of blood product transfusion on short-term lung transplant outcomes. Clin Transplant. 2023;37(6):e14961.

13. Sanaiha Y, Hadaya J, Verma A, Shemin RJ, Madani M, Young N, et al. Morbidity and Mortality Associated With Blood Transfusions in Elective Adult Cardiac Surgery. J Cardiothorac Vasc Anesth. 2023;37(9):1591-8.

14. Stephens LD, Jacobs JW, Adkins BD, Booth GS. Battle of the (Chat)Bots: Comparing Large Language Models to Practice Guidelines for Transfusion-Associated Graft-Versus-Host Disease Prevention. Transfusion Medicine Reviews. 2023;37(3).

15. Wang M, Goldgof GM, Patel A, Whitaker B, Belov A, Chan B, et al. Novel computational methods on electronic health record yields new estimates of transfusion-associated circulatory overload in populations enriched with high-risk patients. Transfusion. 2023;63(7):1298-309.

16. Trutschl M, Cvek U, Trutschl M, editors. Using Artificial Intelligence to Reduce the Risk of Transfusion Hemolytic Reactions. 4th International Conference on Deep Learning Theory and Applications (DeLTA); 2023 2023

Jul 13-14; Rome, ITALY2023.

17. Baucom MR, Price AD, Whitrock JN, Hanseman D, Smith MP, Pritts TA, et al. Association for Academic Surgery Need for Blood Transfusion Volume Is Associated With Increased Mortality in Severe Traumatic Brain Injury. Journal of Surgical Research. 2024;301:163-71.

18. Fung MK, AuBuchon JP, Stephens LD. Classification of posttransfusion adverse events using a publicly available artificial intelligence system. Transfusion. 2024;64(4):590-6.

19. Luo Z, Qin L, Xu S, Yang X, Peng Z, Huang C. Impact of fresh frozen plasma transfusion on mortality in extracorporeal membrane oxygenation. Perfusion-Uk. 2024;39(2):294-303.

20. Zhang H, Zhu Y, Yin X, Sun D, Wang S, Zhang J. Dose-response relationship between perioperative allogeneic blood transfusion and surgical site infections following spinal surgery. Spine Journal. 2024;24(12):2218-23.

21. Kamio T, Ikegami M, Mizuno M, Ishii S, Tajima H, Machida Y, et al. Machine learning-based prediction of bleeding risk in extracorporeal membrane oxygenation patients using transfusion as a surrogate marker. Transfusion. 2025;65(6):1051-60.

22. Okumura K, Dhand A, Misawa R, Sogawa H, Veillette G, Nishida S. Potential Association of Blood Transfusion in Deceased Donors With Outcomes of Liver Transplantation in the United States. Journal of Surgical Research. 2024;300:477-84.

23. Tschoellitsch T, Moser P, Maletzky A, Seidl P, Bock C, Roland T, et al. Potential Predictors for Deterioration of Renal Function After Transfusion. Anesth Analg. 2024;138(3):645-54.

24. Portela GT, Ducrocq G, Bertolet M, Alexander JH, Goodman SG, Glynn S, et al. Individualized transfusion decisions to minimize adverse cardiovascular outcomes in patients with acute myocardial infarction and anemia. American Heart Journal. 2025;282:146-55.
